# Assessing Thermodynamic Selectivity of Solid-State Reactions for the Predictive Synthesis of Inorganic Materials

**DOI:** 10.1021/acscentsci.3c01051

**Published:** 2023-10-16

**Authors:** Matthew J. McDermott, Brennan C. McBride, Corlyn E. Regier, Gia Thinh Tran, Yu Chen, Adam A. Corrao, Max C. Gallant, Gabrielle E. Kamm, Christopher J. Bartel, Karena W. Chapman, Peter G. Khalifah, Gerbrand Ceder, James R. Neilson, Kristin A. Persson

**Affiliations:** † Materials Sciences Division, Lawrence Berkeley National Laboratory, Berkeley, California 94720, United States; ‡ Department of Materials Science and Engineering, University of California, Berkeley, California 94720, United States; § Department of Chemistry, Colorado State University, Fort Collins, Colorado 80523, United States; ∥ Department of Chemistry, Stony Brook University, Stony Brook, New York 11794, United States; ⊥ Department of Chemical Engineering and Materials Science, University of Minnesota, Minneapolis, Minnesota 55455, United States; # Chemistry Division, Brookhaven National Laboratory, Upton, New York 11973, United States; ∇ Molecular Foundry, Lawrence Berkeley National Laboratory, Berkeley, California 94720, United States

## Abstract

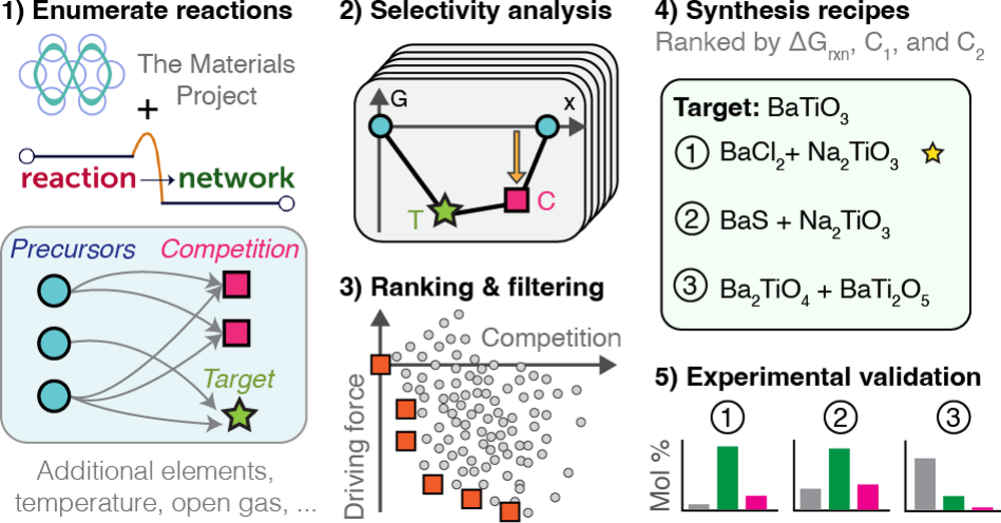

Synthesis is a major challenge in the discovery of new inorganic materials. Currently, there is limited theoretical guidance for identifying optimal solid-state synthesis procedures. We introduce two selectivity metrics, primary and secondary competition, to assess the favorability of target/impurity phase formation in solid-state reactions. We used these metrics to analyze 3520 solid-state reactions in the literature, ranking existing approaches to popular target materials. Additionally, we implemented these metrics in a data-driven synthesis planning workflow and demonstrated its application in the synthesis of barium titanate (BaTiO_3_). Using an 18-element chemical reaction network with first-principles thermodynamic data from the Materials Project, we identified 82985 possible BaTiO_3_ synthesis reactions and selected 9 for experimental testing. Characterization of reaction pathways via synchrotron powder X-ray diffraction reveals that our selectivity metrics correlate with observed target/impurity formation. We discovered two efficient reactions using unconventional precursors (BaS/BaCl_2_ and Na_2_TiO_3_) that produce BaTiO_3_ faster and with fewer impurities than conventional methods, highlighting the importance of considering complex chemistries with additional elements during precursor selection. Our framework provides a foundation for predictive inorganic synthesis, facilitating the optimization of existing recipes and the discovery of new materials, including those not easily attainable with conventional precursors.

## Abstract 2

Selectivity analysis of massive inorganic chemical reaction networks identifies alternative synthesis recipes for solid-state materials (e.g., BaTiO_3_) that outperform conventional approaches.

## Introduction

The predictive synthesis of inorganic materials remains a grand challenge in modern chemistry and materials science.^1^ Unlike organic synthesis, which is often described via discrete reaction steps or mechanisms, inorganic materials synthesis reactions cannot be deconstructed into elementary steps,^2,3^ hindering the analogous development of retrosynthetic analysis techniques^4^ and computer-aided synthesis planning.^5^ This lack of successful mechanistic models has made the synthesis of predicted new materials a critical bottleneck in high-throughput computational materials design efforts,^6^ with many proposed materials having yet to be successfully synthesized.^7−9^

**Figure 1:**
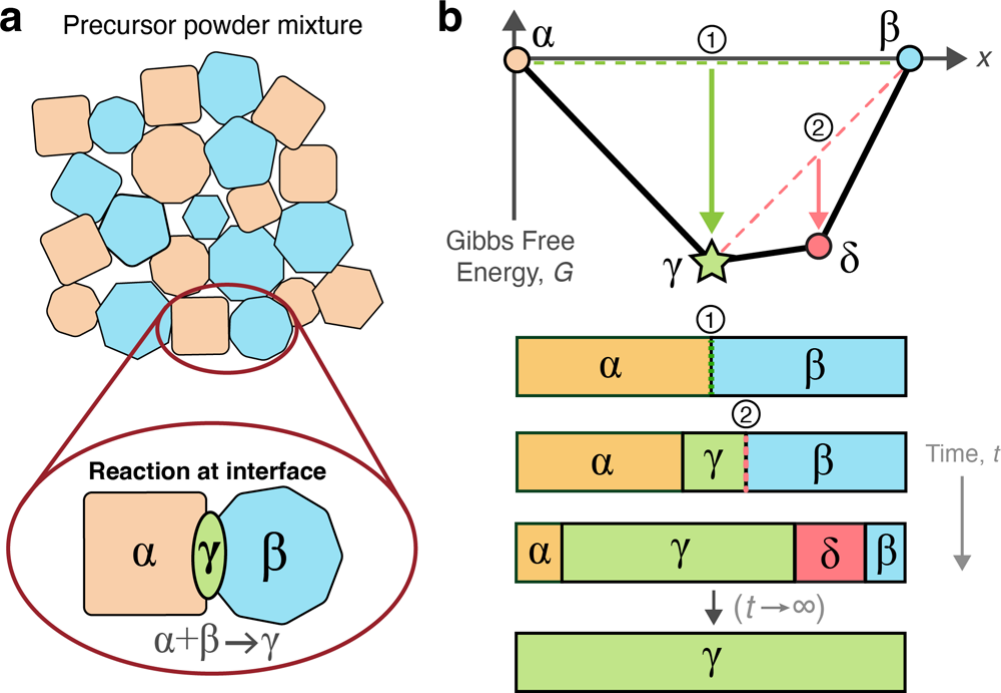
Modeling chemical reactions at heterogeneous solid interfaces in a binary/quasibinary chemical system. (a) Cartoon model of a powder reaction between the hypothetical precursors: α (orange) and β (blue). The nucleation of a new phase, γ (green), occurs at the α|β interface according to the reaction α + β → γ. (b) Possible reaction pathway for the powder system in which a secondary reaction of the equilibrium phase (γ) yields an impurity phase, δ (red). The interface reaction hull (top) shows available interfacial reactions and their corresponding Gibbs free energies, *G*, and mixing ratios, *x*. The one-dimensional spatial model (bottom) shows reaction steps beginning from an equal mixture of α and β. The impurity phase, δ, may be kinetically retained in a local equilibrium state; however, with infinite time, the system should approach the global equilibrium state composed entirely of γ.

While there are numerous inorganic synthesis methods (e.g., hydrothermal, mechanochemical, sol–gel, intercalation, etc.),^10^ we limit the scope of this work to bulk solid-state synthesis via powder reactions. This choice has been motivated by the straightforward and scalable nature of working with bulk powders, which makes solid-state synthesis suitable for applications ranging from one-off laboratory synthesis to industrial mass manufacturing. In powder reactions, product formation proceeds via nucleation and growth at interfacial contact areas in the powder mixture (Figure 1a).^11^ The equilibrium phases of the reacting system can be predicted by constructing a convex hull in free energy and composition space, where the composition axis is a mixing ratio between the two precursor compositions (Figure 1b).^12^ Here, we calculate the convex hull exclusively using normalized compositions and energies (i.e., on a per-atom basis). This construction, which we refer to herein as the “interface reaction hull”, is a subsection of the compositional phase diagram for binary systems and a “quasibinary” two-dimensional slice of the full phase diagram for chemical systems with three or more elements. The exact product species, and the sequence in which they appear, cannot be predicted with thermodynamics alone; to do so requires intimate knowledge of the kinetic rates of all physically feasible reactions. However, a commonly adopted theoretical simplification assumes that the reaction product(s) with the most negative *pairwise* reaction energy will be the first to nucleate and grow as a powder mixture is heated.^11,13^ This hypothesis is based on two principles: (1) the random packing of solid crystallites results in very few locations where three or more particles are simultaneously in contact, and (2) the activation energy barrier to nucleation scales inversely with free energy as . The surface energy, γ, is particularly important when comparing the feasibility of reactions with similar energies.^14^ Despite this, many solid-state reactions are likely not nucleation-limited but rather *transport-limited* due to the relative sluggishness of solid-state diffusion and the often large driving forces of these reactions (10–100 kJ/mol).^15^

The complex interplay between thermodynamics and kinetics makes solid-state synthesis prone to the unpredictable and undesirable formation of impurity phases.^13^ A classic example of a nonselective synthesis is that of the prototypical multiferroic bismuth ferrite, BiFeO_3_, via the standard reaction from binary oxides: Bi_2_O_3_ + Fe_2_O_3_ → 2 BiFeO_3_. This reaction typically yields impurity phases Bi_2_Fe_4_O_9_ and Bi_25_FeO_39_, which are challenging to isolate and remove.^16,17^ Unfortunately, the presence of an impurity phase is difficult to predict *a priori* and is typically attributed to “kinetic” factors or changes in phase equilibria related to precursor purity, morphology, volatility, or processing conditions. To optimize the performance of solid-state reactions and maximize conversion to the desired target, the experimentalist frequently relies on intuition and heuristic rules to choose the (1) precursor compositions (typically off-the-shelf binary phases such as carbonates, oxides, etc.), (2) grinding/milling protocol, (3) synthesis annealing temperature, (4) synthesis atmosphere (e.g., vacuum, flowing O_2_), (5) synthesis time, and (6) cooling protocol. Heuristics include well-known rules such as Tamman’s rule for estimating reaction onset temperature (i.e., two-thirds the melting temperature of the precursor with the lowest melting point),^18^ as well as “chemical intuition” or human-biased experimental protocols (e.g., selecting synthesis times based on common increments, such as 4 h, 8 h, etc.).^19^ Unfortunately, these heuristics may be insufficient to achieve successful synthesis on the first attempt(s), necessitating follow-up experiments that can be time-intensive and costly. In the worst cases, human-biased heuristics lead to lower success rates than randomly generated experimental protocols.^20^

The *a priori* calculation of reaction selectivity in solid-state synthesis permits the ranking of synthesis approaches based on their thermodynamic likelihood of success, thereby circumventing the current time-consuming trial-and-error (Edisonian) approach. The assessment of reaction selectivity is particularly relevant in the proposal of optimal synthesis precursors;^21^ in several cases, improved navigation of the phase diagram was shown to lead to a more practical synthesis.^11,22−25^ However, no solid-state reaction selectivity metric has been formally established. In recent work,^14^ Aykol et al. demonstrated a computational workflow for ranking solid-state synthesis reactions by two performance metrics: (1) a catalytic nucleation barrier factor incorporating structural similarity and epitaxial matching and (2) the number of known competing phases. These metrics perform well in rationalizing successful syntheses in the literature but lack generality; for example, the nucleation metric is derived assuming all reactions are nucleation-limited, which, as discussed, is not true for many solid-state reactions. Additionally, while a metric based on the total *number* of competing phases is significant, as it hints at a measure of reaction selectivity; such a scheme does not account for the *relative* stability of these competing phases. A count-based selectivity metric is also biased by how many phases are known to exist at the present time and the extent to which various structural configurations (e.g., disordered or defective phases) have been enumerated within the data.

In this work, we address the longstanding issue of assessing the selectivity of solid-state reactions by deriving two complementary thermodynamic metrics measuring the degree of phase competition from the interface reaction model. We incorporate these competition metrics into a computational synthesis planning workflow for identifying and ranking synthesis reactions, which builds upon the high-throughput reaction enumeration tools we previously developed for constructing solid-state chemical reaction networks^26^ from large materials databases such as the Materials Project.^27^ Our selectivity metrics, computational workflow, literature analysis, and experimental findings yield a framework for generating more optimal and efficient solid-state synthesis routes, providing a foundation for the predictive synthesis of inorganic materials. The suggestion of nonstandard precursors, particularly those involving additional elements beyond those in the target composition, expands the synthetic capabilities of the solid-state approach.

## Results and Discussion

### Derivation of Selectivity Metrics

#### The Interface Reaction Hull

To construct the interface reaction hull (Figure 1b), one begins with thermodynamic data for the reacting system (i.e., a set of relevant phases and their compositions and energies). In this work, we acquire formation enthalpies, Δ*H*_f_, from the Materials Project database^27^ and extend them to Gibbs free energies of formation, Δ*G*_f_(*T*), through the use of a prior machine learning model^28^ and supplemental experimental thermochemistry data^29^ (see Methods). For systems with two elements, the interface reaction hull is equivalent to the binary compositional phase diagram, where each vertex represents a single phase. However, for systems with three or more elements, the nonprecursor vertices include both single phases and mixtures of phases. These mixtures are stoichiometric combinations of phases representing the products of balanced reactions of the precursors. The balanced reactions can be determined via (1) computing slices of the full compositional phase diagram along the tie-line connecting the precursors^12^ or (2) combinatorial reaction enumeration.^26^ More specifically, the maximum number of products for a particular vertex is one less than the number of elements in the system. The interface reaction hull is thus generalized such that all nonprecursor vertices correspond to reactions with coordinates given by the atomic mixing ratio of precursors, *x*, and the Gibbs free energy of the reaction, Δ*G*_rxn_. This model can be further generalized to environmental conditions other than fixed temperature and pressure by constructing the hull with the appropriate thermodynamic potential. For example, in open systems, one would use the grand potential energy, Φ. Note that in these systems, the hull vertices may include additional open elemental reactants/products (e.g., O_2_) that do not factor into the determination of *x*.

As a model for solid-state reactions, the interface reaction hull construction also rationalizes the formation of impurity phases. To demonstrate this, we revisit the binary system in Figure 1. In this system, the target phase (γ) is predicted to form first because it is the phase with the highest driving force of formation (most negative Δ*G*_rxn_), irrespective of the average composition of the total system.^12^ When the γ phase forms, however, it also introduces two additional interfaces: α|γ and γ|β. These secondary interfaces can be modeled via the construction of new interface reaction hulls or, more simply, by splitting the original hull into two subsections (i.e., to the left and the right of the target). Figure 1b suggests that the γ|β interface should produce an impurity phase via the exergonic reaction γ + β → δ. Hence, the full conversion of reactants to the target phase is impeded while δ persists. In a “one-dimensional” solid-state reaction (Figure 1b, bottom), a local thermodynamic equilibrium may be achieved when the system reaches a state in which all stable product phases on the interface reaction hull have formed and the growth of the product layer(s) slows down until it ceases entirely. This situation has been observed in previous experimental studies on diffusion couples.^30,31^ This observed mixture of products may be kinetically “stable” (i.e., with locally stable interfaces), but it is not the global equilibrium state of the system. Rather, the global equilibrium state is the combination of phases that minimizes the free energy given the composition of the entire powder mixture; in Figure 1b, this corresponds to entirely γ. In powder reactions, access to the global equilibrium state is often provided by regrinding steps in which new interfaces are exposed and mixed to facilitate complete conversion to the equilibrium products. However, in reacting systems with significant phase competition and slow transport, high temperatures and long heating times may be necessary but impractical; pure target synthesis may not be achievable if the desired products are unstable at high temperatures. These situations can be avoided entirely by proposing alternative precursors that are more selective (i.e., those with interface reaction hulls containing few to no competing phases).

#### Measuring Phase Competition

**Figure 2:**
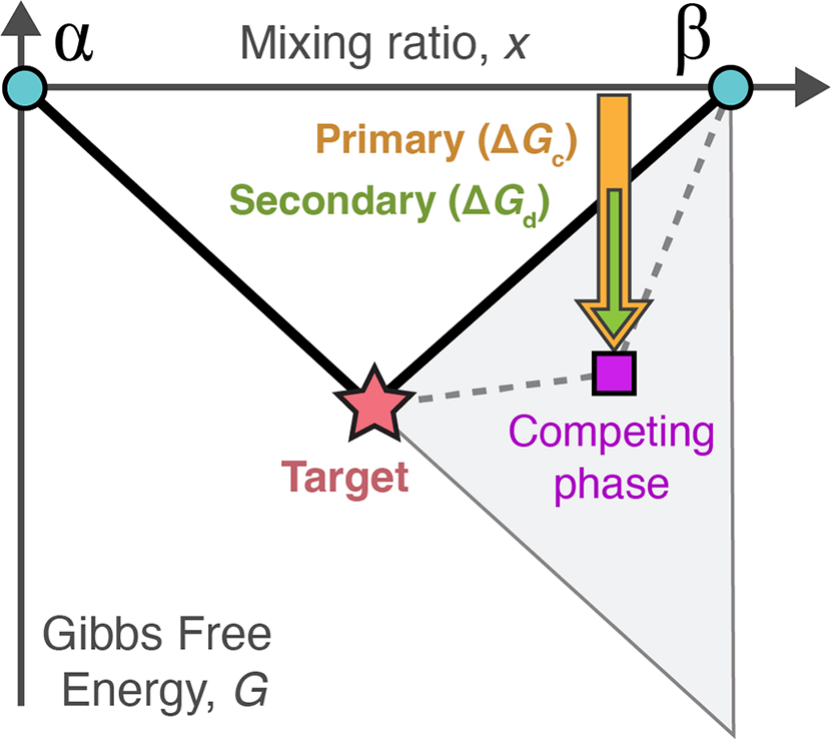
Origin of primary and secondary competition in solid-state reactions. In this simple interface reaction hull for a binary (two-element) system, two interface reactions can form a competing phase (magenta square). The primary reaction (yellow arrow) occurs at the interface between the two precursors α and β, whereas the secondary reaction (green arrow) occurs between the target phase (pink star) and the remaining β precursor, leading to a smaller driving force (arrow length). The coordinates of the competing phase, which must lie within the illustrated bounds (gray triangle) if the target phase is to be stable, determine the relative values of the primary and secondary reaction energies.

To predict the thermodynamic selectivity of a solid-state reaction, we propose two complementary metrics for assessing phase competition using the interface reaction hull: primary (*C*_1_) and secondary (*C*_2_) competition. Although both metrics measure the relative energetic favorability of competing reactions, they model different mechanisms for impurity formation. The primary competition measures the favorability of competing reactions of the original precursors, while the secondary competition measures the favorability of *subsequent* competing reactions between the precursors and target phase(s). The origin of the two competition mechanisms is illustrated in Figure 2.

Primary competition, *C*_1_, is measured via calculation of the relative thermodynamic advantage of the most exergonic competing reaction from the original precursors, as assessed through an energy difference:
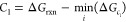
1

Here, Δ*G*_rxn_ is the energy of the target synthesis reaction, and Δ*G*_*c*_*i*__ are the energies of possible competing reactions from the precursors. Lower *C*_1_ values are favorable and result in more selective target formation. When *C*_1_ is positive, the target reaction is less energetically favorable than the competing reaction with the greatest driving force (most negative energy), suggesting that a competing phase is likely to form. On the other hand, when *C*_1_ is negative, the target reaction is predicted to have the greatest driving force of any reaction on the hull. By considering only the single most competitive reaction, this functional form avoids the aforementioned bias related to using the total *number* of competing reactions. When no exergonic competing reactions are predicted for an interface, the competing reaction energy term is assigned a value of zero, representing the scenario in which the precursors do not react (e.g., α → α). This results in the limiting condition *C*_1_ ≥ Δ*G*_rxn_.

Secondary competition, *C*_2_, assesses the favorability of impurity phase formation via secondary reactions between the target and precursor(s). This metric is important and distinct from primary competition because it measures the relative stability of the products of the target synthesis reaction with respect to decomposition into the competing phase(s). Their relative stability can be measured by computing the “inverse distance to the hull” (Figure 2), which for systems with one competing phase is equivalent to the secondary reaction energy, Δ*G*_*d*_, at the precursor–target interface.

**Figure 3:**
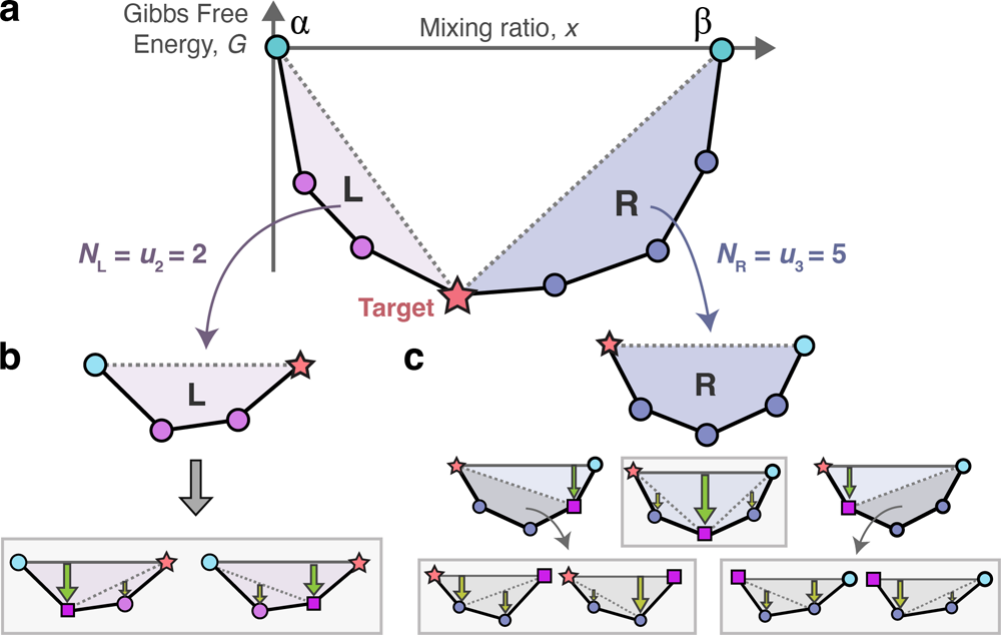
Secondary reaction sequences in an interface reaction hull. (a) The hull is divided into two subsections to the left (L) and right (R) of the target, representing the two additional precursor–target interfaces. (b, c) Secondary reaction sequences on either side of the target, with gray boxes highlighting the final reaction sequences. The recursive, binary nature results in the number of unique sequences, *N*(*n*), following the Catalan numbers *u*_*n*_.

In an interface reaction hull with several competing phases, a sequence of multiple secondary reactions may occur (Figure 3). When the target phase is formed, it introduces two new precursor–target interfaces that divide the hull into subsections to the left and right of the target. A secondary reaction may occur in either subsection, exposing another two interfaces. If, at either interface, there is a remaining driving force to form additional competing phases, then this process may continue in a recursive fashion until all possible secondary reactions have occurred. There are multiple ways to draw a feasible secondary reaction sequence (Figure 3b,c). Consider a particular secondary reaction sequence indexed *j*. This sequence has a total energy given by the sum of the energies of its *n* steps
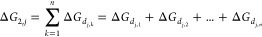
2where the number of reaction steps (*n*) in the sequence also equals the number of nonreactant (interior) vertices in the hull subsection.

If every secondary reaction step is required to be the one with the minimum energy (i.e., largest driving force), then only one unique reaction sequence exists in the left and right hull subsections. However, one must consider the situation where the minimum-energy principle does not hold due to kinetic limitations; this applies in particular to hulls where all secondary reactions have similar magnitude driving forces or a particular phase is kinetically limited from forming, perhaps due to an overall small driving force. Therefore, we must consider the alternative secondary reaction sequences shown in Figure 3b,c. These alternative sequences are not necessarily less favorable; while each alternative sequence may feature a first reaction step with a smaller driving force (i.e., small Δ*G*_*d*_*j*,1__), the latter steps may have larger magnitude energies, resulting in comparable total energy (Δ*G*_2,*j*_) for the particular sequence.

To encompass all combinatorial possibilities in our estimation of secondary competition, we choose to compute the mean total energy of all feasible secondary reaction sequences:
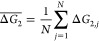
3

Determining the total number of unique secondary reaction sequences, *N*, is mathematically equivalent to calculating the total number of full binary trees with *n* interior nodes, which yields the Catalan number sequence, *u*_*n*_ = 1, 1, 2, 5, 14, 42, 132, 429, ... (*n* = 0, 1, 2, ...).^32^ Using this connection to the Catalan numbers, we developed a nonrecursive algorithm for calculating  that is significantly faster than the equivalent recursive solution (see Methods).

Finally, we formulate the *C*_2_ metric such that it accounts for all possible reaction sequences in either hull subsection to the left (L) and right (R) of the target phase:
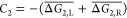
4

The negative factor is included so that a lower *C*_2_ value corresponds to a more favorable selectivity. Because our definition of a secondary reaction assumes that Δ*G*_*d*_ ≤ 0, the secondary competition metric obeys the limiting behavior: *C*_2_ ≥ 0.

We note that both *C*_1_ and *C*_2_ implicitly assume that the target phase is thermodynamically stable (“on the hull”) under the conditions for which the equilibrium phase diagram is derived. However, the competition metrics are still calculable for a metastable phase by manually decreasing its energy until it becomes stable.

### Application to Experimental Literature

**Figure 4:**
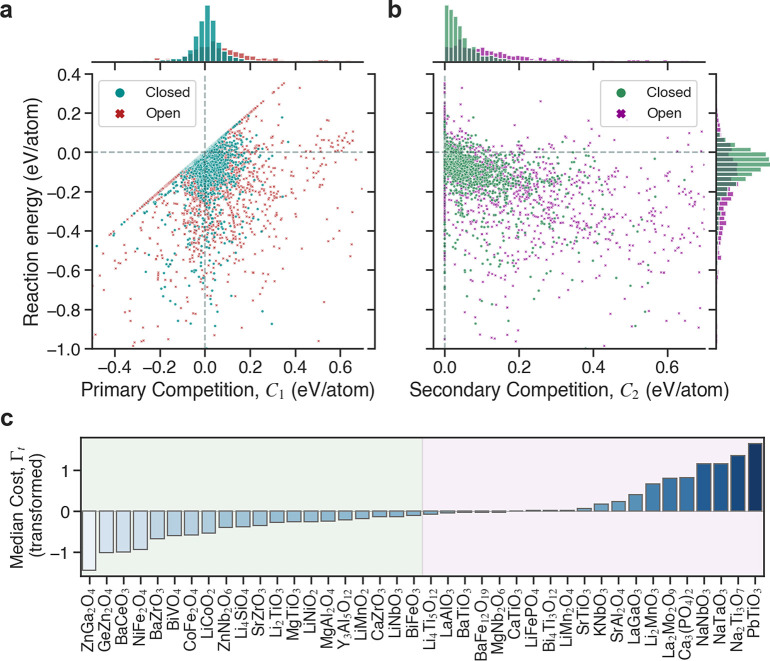
Thermodynamic analysis of synthesis recipes in the experimental solid-state literature. Synthesis maps of 3520 literature reactions from the Kononova et al. data set, plotted on a shared axis of reaction energy, Δ*E*_rxn_, and independent axes of (a) primary competition, *C*_1_, and (b) secondary competition, *C*_2_. These selectivity metrics are constrained by their lower bounds: *C*_1_ = Δ*E*_rxn_ (diagonal parity line) and *C*_2_ = 0. (c) Median transformed cost (Γ_t_) rankings of synthesis recipes for each of the 40 most popular targets in the data set. The shading marks the targets with average recipes below (green) or above (pink) the median Γ_t_ of all experiments in the data set (−0.089). Selected recipes are discussed in Tables 1 and 2. The full data set is provided in the Supporting Information.

Using the competition metrics *C*_1_ and *C*_2_, we now assess the selectivities of solid-state reactions previously reported in the experimental literature and use these to rank synthesis recipes by their predicted thermodynamic optimality. Reaction energies and competition metrics were calculated for 3520 unique experiments reported in the text-mined solid-state reaction literature data set by Kononova et al.^33^ Each unique experiment corresponds to a particular balanced reaction, maximum synthesis temperature, and atmospheric environment (e.g., air, flowing O_2_, etc.). We modeled all reactions containing up to two solid precursors and an optional gaseous reactant (see Methods). Reactions that were reported with no particular atmospheric environment are denoted as “closed” and modeled with Gibbs free energies (Δ*G*_rxn_), while those with a defined environment are denoted as “open” and modeled using grand potential energies (ΔΦ_rxn_). The results of these calculations are shown in Figure 4a,b.

The synthesis “maps” (Figure 4a,b) allow one to identify favorable reactions by comparing the relative weights of the three reaction metrics: reaction energy (Δ*E*_rxn_), primary competition (*C*_1_), and secondary competition (*C*_2_). Thermodynamically optimal reactions are ones that minimize all three metrics, resulting in placement in the lower left region of each plot. According to our calculations, many reactions reported in the literature are predicted to be energetically favorable and selective. Approximately 17.3% of reactions have no exergonic competing reactions on the interface reaction hull (i.e., they are on the bounding line *C*_1_ = Δ*E*_rxn_), and 23.0% of reactions have negligible secondary competition (*C*_2_ ≤ 0.001 eV/atom). Assuming the literature reactions are experimentally feasible, one would expect all calculated reaction energies to be negative. Our thermodynamic modeling captures this within reasonable error: 82.8% of reactions have a negative reaction energy, and 97.0% of reactions have Δ*E*_rxn_ ≤ 0.1 eV/atom. Of the reactions with positive energies, most (87.1%) contain one or more common gases: O_2_ (85.1%), CO_2_ (48.8%), or H_2_O (8.4%). A major source of error in our reaction energy calculations is likely the disagreement between the assumed and actual gas partial pressures; it is often challenging to model the actual environmental conditions of synthesis, as it requires that they be both (1) accurately reported and (2) correctly extracted from the text. Another known source of error is systematic challenges in estimating GGA-calculated formation energies of carbonate compounds.^14^ However, this has been addressed and partially mitigated with a fitted energy correction (see Methods).

To quantitatively assess thermodynamic optimality, we follow a approach similar to that of prior works^24,26^ and define a cost function, Γ, that combines the driving force and reaction selectivity evaluated at a particular set of conditions (i.e., temperature and atmosphere). In this work, we opt to use a simple linear weighted summation of the reaction’s energy and competition scores
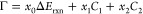
5where Δ*E*_rxn_ is the reaction energy (either Δ*G*_rxn_ or ΔΦ_rxn_) and *x*_0_, *x*_1_, and *x*_2_ are user-defined weights for each parameter. Due to the different scaling of each parameter, we find that a nonequal weighting of *x*_0_ = 0.10, *x*_1_ = 0.45, and *x*_2_ = 0.45 produces reasonably diverse results that do not significantly favor one parameter over another. We note that this selection is arbitrary and subject to further optimization.

Unfortunately, closed and open reactions cannot be rigorously compared due to their different energy scales (i.e., Gibbs free energies vs grand potential energies). This is further evidenced by differences in the reaction metric distributions (Figure 4a,b). Since no ideal solution exists for ranking and comparing reactions under different environmental conditions, we transform the cost function to account for the energy scale difference. To do this, we apply a power transformation to the cost distributions for the closed and open reactions, resulting in monotonically transformed costs, Γ_t_, whose distributions are closer to standard normal distributions (Figure S1). This new variable facilitates a fairer comparison between closed and open reactions, allowing for a more realistic ranking of synthesis recipes.

Figure 4c shows the median values of Γ_t_ calculated from the synthesis recipes of the 40 most popular targets in the literature data set (i.e., those with the most reactions extracted from the text). Given the extent of coverage of our thermodynamic data, we limit our analysis to only the targets for which we have at least five recipes successfully calculated (see Methods). In the following sections, we select several targets with costs below and above the median Γ_t_ value, analyzing the factors leading to their optimal and suboptimal synthesis recipes, respectively.

#### Optimal Literature Recipes

Of the 40 most popular targets in the literature data set (Figure 4c), 20 have synthesis recipes with an average cost value (Γ_t_) below the data set’s median (−0.089), indicating generally favorable thermodynamic optimality. A selection of these targets and their highest/lowest-ranked recipes are provided in Table 1, along with other selected reactions of interest. DOIs and raw (untransformed) costs for each reaction are provided in the Supporting Information.

**Table 1:** Thermodynamic Analysis of Optimal Experimental Synthesis Recipes for Selected Popular Targets in the Literature^a^

target	rank	reaction	temp (°C)	energy (eV/at)	open	*C*_1_ (eV/at)	*C*_2_ (eV/at)	Γ_t_ (au)
ZnGa_2_O_4_	1	ZnO + Ga_2_O_3_ → ZnGa_2_O_4_	1400	–0.218	O	–0.218	0.000	–1.622
	12	ZnO + Ga_2_O_3_ → ZnGa_2_O_4_	1200	–0.088		–0.088	0.000	–0.969
	17	ZnO + Ga_2_O_3_ → ZnGa_2_O_4_	500	–0.067		–0.067	0.000	–0.818
								
BiVO_4_	1	0.5 Bi_2_O_3_ + 0.5 V_2_O_5_ → BiVO_4_	600	–0.529	O	–0.210	0.171	–1.156
	5	0.5 Bi_2_O_3_ + 0.5 V_2_O_5_ → BiVO_4_	500	–0.169		–0.061	0.034	–0.713
	12	Bi + 0.5 V_2_O_5_ + 0.75 O_2_ → BiVO_4_	600	–1.860	O	0.801	4.489	2.412
								
CoFe_2_O_4_	1	CoO + Fe_2_O_3_ → CoFe_2_O_4_	1250	–0.088	O	–0.062	0.000	–0.877
	3	0.3333 Co_3_O_4_ + Fe_2_O_3_ → CoFe_2_O_4_ + 0.1667 O_2_	950	–0.058	O	–0.018	0.011	–0.662
	11	Co + 2 Fe + 2 O_2_ → CoFe_2_O_4_	1400	–1.738	O	0.223	2.957	2.111
	14	CoCO_3_ + Fe_2_O_3_ → CoFe_2_O_4_ + CO_2_	1400	–0.215		0.156	0.370	2.781
								
LiCoO_2_	1	LiOH + 0.3333 Co_3_O_4_ + 0.08333 O_2_ → LiCoO_2_ + 0.5 H_2_O	700	–0.120	O	–0.012	0.000	–0.727
	4	0.5 Li_2_CO_3_ + 0.3333 Co_3_O_4_ + 0.08333 O_2_ → LiCoO_2_ + 0.5 CO_2_	950	–0.061	O	–0.014	0.000	–0.692
	23	0.5 Li_2_CO_3_ + CoCO_3_ + 0.25 O_2_ → LiCoO_2_ + 1.5 CO_2_	300	–0.121	O	0.154	0.357	0.532
	30	LiOH·H_2_O + Co + 0.75 O_2_ → LiCoO_2_ + 1.5 H_2_O	900	–0.382	O	1.001	1.454	1.992
								
Li_4_SiO_4_	1	2 Li_2_CO_3_ + SiO_2_ → Li_4_SiO_4_ + 2 CO_2_	1445	–0.040	O	–0.017	0.000	–0.683
	7	4 LiOH + SiO_2_ → Li_4_SiO_4_ + 2 H_2_O	700	–0.049		–0.004	0.000	–0.412
	10	2 Li_2_CO_3_ + SiO_2_ → Li_4_SiO_4_ + 2 CO_2_	1150	–0.010		–0.005	0.000	–0.366
	18	4 Li + SiO_2_ + O_2_ → Li_4_SiO_4_	800	–2.133	O	0.163	2.830	2.038
								
LiNiO_2_	1	LiOH·H_2_O + NiO + 0.25 O_2_ → LiNiO_2_ + 1.5 H_2_O	480	0.008	O	0.010	0.002	–0.559
	4	0.5 Li_2_O + NiO + 0.25 O_2_ → LiNiO_2_	800	0.032	O	0.058	0.021	–0.344
	6	LiOH + Ni(OH)_2_ + 0.25 O_2_ → LiNiO_2_ + 1.5 H_2_O	770	0.074	O	0.074	0.000	–0.331
	24	0.5 Li_2_O_2_ + NiO → LiNiO_2_	800	–0.035		0.067	0.147	0.973
	27	Li + Ni(OH)_2_ + 0.5 O_2_ → LiNiO_2_ + H_2_O	700	–0.536	O	1.770	3.044	2.396
								
Y_3_Al_5_O_12_	1	1.5 Y_2_O_3_ + 2.5 Al_2_O_3_ → Y_3_Al_5_O_12_	600	–0.071		–0.034	0.000	–0.623
	15	1.5 Y_2_O_3_ + 2.5 Al_2_O_3_ → Y_3_Al_5_O_12_	1727	–0.240	O	–0.051	0.221	–0.260
	36	1.5 Y_2_O_3_ + 5 Al(OH)_3_ → Y_3_Al_5_O_12_ + 7.5 H_2_O	1300	–0.120		–0.007	0.118	0.202
								
BiFeO_3_	1	0.5 Bi_2_O_3_ + 0.5 Fe_2_O_3_ → BiFeO_3_	600	–0.073	O	0.034	0.053	–0.386
	4	0.5 Bi_2_O_3_ + 0.5 Fe_2_O_3_ → BiFeO_3_	100	–0.011		0.000	0.004	–0.312
	38	Bi + 0.5 Fe_2_O_3_ + 0.75 O_2_ → BiFeO_3_	900	–1.285	O	1.087	3.558	2.364

a The ranking of reactions is determined by the transformed cost, Γ_t_. The highest- and lowest-ranked reactions are shown along with selected reactions of interest. Raw costs and DOIs are available in the Supporting Information.

For many targets in Table 1, the conventional reaction involving off-the-shelf binary precursors is predicted to be thermodynamically optimal. For example, in the synthesis of the spinel ZnGa_2_O_4_, the reaction between the binary oxides, ZnO + Ga_2_O_3_ → ZnGa_2_O_4_, is already perfectly selective (*C*_1_ = Δ*E*_rxn_ and *C*_2_ = 0) over all temperatures in the data set. Furthermore, these precursors appear in *all* 17 calculated literature recipes for this target. The favorability of the conventional route seems to apply to several other targets presented here, including BiVO_4_, CoFe_2_O_4_, Y_3_Al_5_O_12_, and BiFeO_3_. Since the conventional precursors for these targets are more favorable than the explored alternatives, we can instead analyze which synthesis *conditions* are most optimal. For CoFe_2_O_4_, higher temperatures in an open oxygen environment appear to be the most favorable. On the other hand, lower temperatures in an open oxygen environment favor the production of BiVO_4_ and BiFeO_3_. Finally, for Y_3_Al_5_O_12_, there appears to be a tradeoff between selectivity (which is optimal at lower temperatures) and driving force (which is optimal at high temperatures); it appears that intermediate temperatures (600 °C) in a closed environment result in the most suitable compromise. We note, however, that there are many reasons to use specific processing conditions outside of the pursuit of target phase purity (e.g., improved density, annihilation of defects, optimization of crystallite sizes, etc.). These alternative reasons may explain some of the variability of conditions reported in the literature.

Interestingly, several targets in Table 1 feature synthesis recipes that appear extremely unfavorable. These often involve elemental precursors, such as Bi + 0.5 V_2_O_5_ + 0.75 O_2_ → BiVO_4_ or Bi + 0.5 Fe_2_O_3_ + 0.75 O_2_ → BiFeO_3_. Referencing the original articles from which these reactions were sourced^34,35^ suggests that these precursors were not actually used, and the reaction’s inclusion in the data set is likely the result of an error in the literature extraction process. This explains some other impractical and highly suboptimal routes in the data set, such as reactions involving alkali-metal precursors (e.g., 4 Li + SiO_2_ + O_2_ → Li_4_SiO_4_).

For other targets such as LiCoO_2_, Li_4_SiO_4_, and LiNiO_2_, there appears to be great variability in precursor selection in the literature. For LiCoO_2_, the optimal synthesis recipe from our calculations is the reaction of LiOH and Co_3_O_4_ in a flowing oxygen environment at low to intermediate temperatures (i.e., 700 °C). The use of Co_3_O_4_ leads to significantly greater performance than recipes using CoCO_3_. Additionally, using LiOH may offer a thermodynamic advantage over Li_2_CO_3_, although with little effect on the reaction selectivity. A similar conclusion applies to the synthesis of Li_4_SiO_4_, although the use of lithium carbonate appears to be more favorable at high temperatures in an open-oxygen environment. LiOH (particularly the monohydrate) also appears to offer some advantage in the synthesis of LiNiO_2_, especially in oxygen at low temperatures (480–600 °C).

#### Suboptimal Literature Recipes

Reactions can still be successful despite high *C*_1_ and *C*_2_ values. However, our thermodynamic assessment suggests that these reactions are suboptimal and likely require some combination of (1) long heating times to promote thermodynamic equilibrium, (2) follow-up regrinding steps, or (3) fine-tuning of temperature and reaction atmosphere. In Table 2, we highlight several popular target materials that are associated with higher-than-average costs for their synthesis recipes.

**Table 2:** Thermodynamic Analysis of Suboptimal Experimental Synthesis Recipes for Selected Popular Targets in the Literature^a^

target	rank	reaction	temp (°C)	energy (eV/at)	open	*C*_1_ (eV/at)	*C*_2_ (eV/at)	Γ_t_ (au)
PbTiO_3_	1	PbCO_3_ + TiO_2_ → PbTiO_3_ + CO_2_	1200	–0.402	O	0.118	0.750	0.960
	2	PbO + TiO_2_ → PbTiO_3_	1100	–0.360	O	0.186	0.694	0.987
	13	PbO + TiO_2_ → PbTiO_3_	800	–0.163		0.136	0.288	2.172
								
Na_2_Ti_3_O_7_	1	2 NaOH + 3 TiO_2_ → Na_2_Ti_3_O_7_ + H_2_O	750	–0.058	O	0.079	0.046	–0.273
	3	Na_2_CO_3_ + 3 TiO_2_ → Na_2_Ti_3_O_7_ + CO_2_	1100	0.009		0.108	0.104	1.025
	8	Na_2_CO_3_ + 3 TiO_2_ → Na_2_Ti_3_O_7_ + CO_2_	80	0.053		0.202	0.200	2.344
								
NaTaO_3_	1	0.5 Na_2_CO_3_ + 0.5 Ta_2_O_5_ → NaTaO_3_ + 0.5 CO_2_	1150	–0.088		0.035	0.085	0.300
	4	0.5 Na_2_CO_3_ + 0.5 Ta_2_O_5_ → NaTaO_3_ + 0.5 CO_2_	1200	–0.233	O	0.285	0.665	1.098
	9	0.5 Na_2_CO_3_ + 0.5 Ta_2_O_5_ → NaTaO_3_ + 0.5 CO_2_	900	–0.194	O	0.441	0.866	1.435
								
Ca_3_(PO_4_)_2_	1	CaCO_3_ + Ca_2_P_2_O_7_ → Ca_3_(PO_4_)_2_ + CO_2_	800	–0.068		–0.013	0.000	–0.494
	3	3 CaCO_3_ + 2 NH_4_H_2_PO_4_ → Ca_3_(PO_4_)_2_ + 3 CO_2_ + 3 H_2_O + 2 H_3_N	1150	–0.199	H	0.078	0.269	0.187
	4	3 CaCO_3_ + 2 (NH_4_)_2_HPO_4_ → Ca_3_(PO_4_)_2_ + 3 CO_2_ + 3 H_2_O + 4 H_3_N	1200	–0.156	N	0.105	0.374	0.463
	10	3 CaO + P_2_O_5_ → Ca_3_(PO_4_)_2_	550	–0.641		–0.007	0.463	1.673
	12	3 CaCO_3_ + 2 (NH_4_)_2_HPO_4_ → Ca_3_(PO_4_)_2_ + 3 CO_2_ + 3 H_2_O + 4 H_3_N	1200	–0.143		0.094	0.336	2.232
								
Li_2_MnO_3_	1	Li_2_CO_3_ + MnO_2_ → Li_2_MnO_3_ + CO_2_	975	–0.096	O	–0.015	0.015	–0.670
	4	2 LiOH + MnO_2_ → Li_2_MnO_3_ + H_2_O	650	–0.092		–0.024	0.010	–0.533
	5	2 LiOH·H_2_O + MnO_2_ → Li_2_MnO_3_ + 3 H_2_O	70	–0.059		0.001	0.016	–0.295
	14	Li_2_CO_3_ + MnCO_3_ + 0.5 O_2_ → Li_2_MnO_3_ + 2 CO_2_	500	–0.196	O	0.262	0.545	0.942
	15	2 LiOH·H_2_O + MnCO_3_ + 0.5 O_2_ → Li_2_MnO_3_ + CO_2_ + 3 H_2_O	450	–0.187	O	0.253	0.554	0.943
	25	2 Li + MnO_2_ + 0.5 O_2_ → Li_2_MnO_3_	700	–1.971	O	0.400	2.399	1.989
								
LiMn_2_O_4_	1	0.5 Li_2_CO_3_ + 2 MnO_2_ → LiMn_2_O_4_ + 0.5 CO_2_ + 0.25 O_2_	700	–0.032	O	0.041	0.051	–0.347
	2	LiOH + 2 MnO_2_ → LiMn_2_O_4_ + 0.5 H_2_O + 0.25 O_2_	1000	–0.052	O	0.031	0.067	–0.343
	12	0.5 Li_2_CO_3_ + Mn_2_O_3_ + 0.25 O_2_ → LiMn_2_O_4_ + 0.5 CO_2_	950	–0.073	O	0.030	0.110	–0.240
	47	0.5 Li_2_CO_3_ + 2 MnCO_3_ + 0.75 O_2_ → LiMn_2_O_4_ + 2.5 CO_2_	400	–0.319	O	0.148	0.488	0.664
	63	0.5 Li_2_O_2_ + Mn_2_O_3_ → LiMn_2_O_4_	800	–0.138		0.121	0.283	2.072
								
LiFePO_4_	1	0.3333 Li_3_PO_4_ + 0.3333 Fe_3_(PO_4_)_2_ → LiFePO_4_	600	–0.020		–0.008	0.002	–0.386
	2	LiPO_3_ + 0.5 Fe_2_O_3_ → LiFePO_4_ + 0.25 O_2_	1200	–0.151	O	0.108	0.120	–0.060
	3	0.3333 Li_3_PO_4_ + 0.3333 Fe_3_(PO_4_)_2_·8H_2_O → LiFePO_4_ + 2.667 H_2_O	800	–0.065		0.000	0.068	0.012
	4	LiOH·H_2_O + Fe(PO_4_)·2H_2_O → LiFePO_4_ + 3.5 H_2_O + 0.25 O_2_	650	–0.111	O	0.062	0.247	0.149
	5	0.5 Li_2_CO_3_ + FePO_4_ → LiFePO_4_ + 0.5 CO_2_ + 0.25 O_2_	700	0.006	O	0.189	0.122	0.207
								
BaTiO_3_	1	BaCO_3_ + TiO_2_ → BaTiO_3_ + CO_2_	1050	–0.056	O	0.075	0.065	–0.230
	16	BaCO_3_ + TiO_2_ → BaTiO_3_ + CO_2_	1100	–0.028		0.025	0.026	–0.045
	37	BaO + TiO_2_ → BaTiO_3_	800	–0.239		0.061	0.168	0.778
	40	BaO + TiO_2_ → BaTiO_3_	1300	–0.225		0.080	0.167	0.916

a The ranking of reactions is determined by the transformed cost, Γ_t_. The highest- and lowest-ranked reactions are shown along with selected reactions of interest. Raw costs and DOIs are available in the Supporting Information.

Our findings suggest that the targets in Table 2 require a more judicious synthesis due to inherently greater competition in their phase space. Many targets are ranked poorly because the conventional reaction is predicted to be suboptimal. This appears to be true for lead titanate (PbTiO_3_), which, on average, has the most suboptimal recipe of any target investigated (Figure 4c). The high *C*_1_ and *C*_2_ values for PbTiO_3_ synthesis seem to be almost entirely related to the instability of the PbO precursor, with respect both to decomposition and to the formation of a solid-solution phase. In our calculations, the latter manifests as the predicted stabilization of a theoretical Pb_15_TiO_17_ phase. Our modeling of PbO instability supports experimental observations suggesting that the volatility of PbO at higher temperatures results in a challenging PbTiO_3_ synthesis.^36^ The use of PbCO_3_ as an alternative appears to perform similarly, albeit with a slightly more favorable driving force and lower *C*_1_.

For Ca_3_(PO_4_)_2_, Li_2_MnO_3_, and LiFePO_4_, the highest-ranked synthesis recipes appear to be nearly thermodynamically optimal already (i.e., low *C*_1_ and *C*_2_). The optimal recipe for Ca_3_(PO_4_)_2_ is the reaction of stoichiometric amounts of CaCO_3_ and Ca_2_P_2_O_7_ at moderate temperatures (800 °C) in a closed environment. For Li_2_MnO_3_, the reaction of Li_2_CO_3_ and MnO_2_ in open-oxygen environments at moderately high temperatures (900–975 °C) is favorable, and the use of LiOH in a closed environment at lower temperatures (650 °C) appears to be similarly advantageous.^37^ Finally, for the synthesis of LiFePO_4_, the use of Li_3_PO_4_ and Fe_3_(PO_4_)_2_ in a closed environment at lower temperatures (600 °C)^38^ appears to be highly selective and greatly outperforms the other recipes analyzed in the literature data set.

For the remaining targets Na_2_Ti_3_O_7_, NaTaO_3_, LiMn_2_O_4_, and BaTiO_3_, even the highest-ranked literature synthesis recipes are theoretically suboptimal, which suggests that these materials are suitable candidates for future synthesis optimization efforts. Recipes for Na_2_Ti_3_O_7_ generally feature poor driving forces and low selectivities; however, using NaOH as an alternative precursor mitigates this some. All NaTaO_3_ recipes in our data set used the same precursors, but those with open-oxygen environments resulted in substantially higher phase competition. LiMn_2_O_4_ synthesis appears to follow trends similar to those discussed previously for LiMn_2_O_3_; the lithium carbonate route in open air appears to be the most optimal of the recipes explored, despite a somewhat low driving force and moderate competition.^39^ The use of a MnCO_3_ precursor is not recommended due to greatly decreased selectivity. Finally, for BaTiO_3_, the conventional BaCO_3_ route at high temperatures (1050 °C) and open-oxygen conditions appears to be the most favorable; however, with this route, the driving force is still somewhat low and features a moderate amount of phase competition. The BaTiO_3_ system will be examined extensively in the following sections as an experimental case study for assessing reaction selectivity.

The literature reaction data reveal an inherent tradeoff between driving force and selectivity. Reactions with *only* elemental precursors (e.g., Li metal, O_2_ gas) typically have significantly greater driving forces but also much higher competition scores (i.e., lower selectivity), with median values of Δ*G*_rxn_ = −0.272, *C*_1_ = 0.016, and *C*_2_ = 0.112 eV/atom for the closed reactions. For reactions with at least one binary (two-element) precursor, these values shift to Δ*G*_rxn_ = −0.037, *C*_1_ = 0.006, and *C*_2_ = 0.020 eV/atom. In other words, sourcing an element from a precursor with preformed bonds yields an interface reaction hull representing a more selective slice of the phase diagram, but at the expense of sacrificing some of the available reaction energy. Fortunately, this tradeoff is not universal and can be circumvented by considering more complex precursor chemistries containing additional elements other than those in the target composition. For example, in oxide synthesis, the use of hydroxides, carbonates, and salts (as in metathesis reactions) often permits the formation of thermodynamically favorable byproducts that increase the reaction’s driving force and selectivity. The use of additional elements beyond those found in the target composition has been dubbed “hyperdimensional chemistry”^40^ due to its connection to phase diagram geometry; adding a new compositional axis greatly increases the number of ways the phase space can be sliced, allowing one to thermodynamically “short cut” otherwise unavoidable competing impurity phases. However, not all elemental precursors are unselective, and the necessity of these alternative routes depends on the degree of phase competition in the chemical system of interest. For example, it is typically favorable to synthesize a binary target comprised of elements commonly existing in only a single oxidation state; this is exemplified by the reaction Cd + Te → CdTe, which has both perfect selectivity (*C*_1_ = −0.576, *C*_2_ = 0.000 eV/atom) and a very large driving force (Δ*G*_rxn_ = −0.576 eV/atom) at 800 °C.

### Case Study: Synthesis of Barium Titanate (BaTiO_3_)

To investigate whether our reaction selectivity metrics accurately describe impurity formation, we designed and performed experiments testing the influence of precursor selection on the reaction pathway observed during solid-state synthesis. To suggest these experiments, we developed a computational synthesis planning workflow that integrates our proposed selectivity metrics with previous methods for computing solid-state reaction networks.^26^ The workflow has five user inputs: (1) target composition, (2) additional elements, (3) temperature, (4) thermodynamic stability cutoff (i.e., maximum *E*_hull_), and (5) chemical potential of an open element, if any. The workflow returns a ranked list of possible synthesis reactions based on the cost function implemented in eq 5.

We selected the ferroelectric barium titanate (BaTiO_3_) as a case study system for testing our predictions. In addition to being a technologically important and well-studied material in the literature, BaTiO_3_ is an ideal target for investigating the thermodynamic selectivity of solid-state reactions due to the high number of competing phases in the Ba–Ti–O chemical system (Figure S2). Conventional routes from binary oxides are well-known to proceed through intermediates,^41^ and at least 12 unique ternary compositions have been experimentally observed, including the compositional neighbors BaTi_2_O_5_ and Ba_2_TiO_4_, which frequently appear during synthesis (the latter in particular). Other commonly observed minor impurities include Ba_4_Ti_13_O_30_, BaTi_4_O_9_, and Ba_6_Ti_17_O_40_. Additional compositions in this space, such as Ba_2_Ti_9_O_20_, BaTi_5_O_11_, Ba_4_Ti_12_O_27_, and Ba_2_Ti_6_O_13_, have been previously synthesized but are less commonly observed as intermediates or impurity phases.^41^ Another motivation for selecting BaTiO_3_ is the kinetic accessibility of its chemical system; even with such a high number of competing Ba–Ti–O phases, the solid-state synthesis of barium titanate is generally considered to be facile. The ease with which Ba–Ti–O phases can be synthesized suggests that kinetic factors do not significantly divert reaction outcomes from those that are thermodynamically favorable, providing greater justification for our attempts at assessing the likelihood of intermediate/impurity formation through a purely thermodynamic lens.

**Figure 5:**
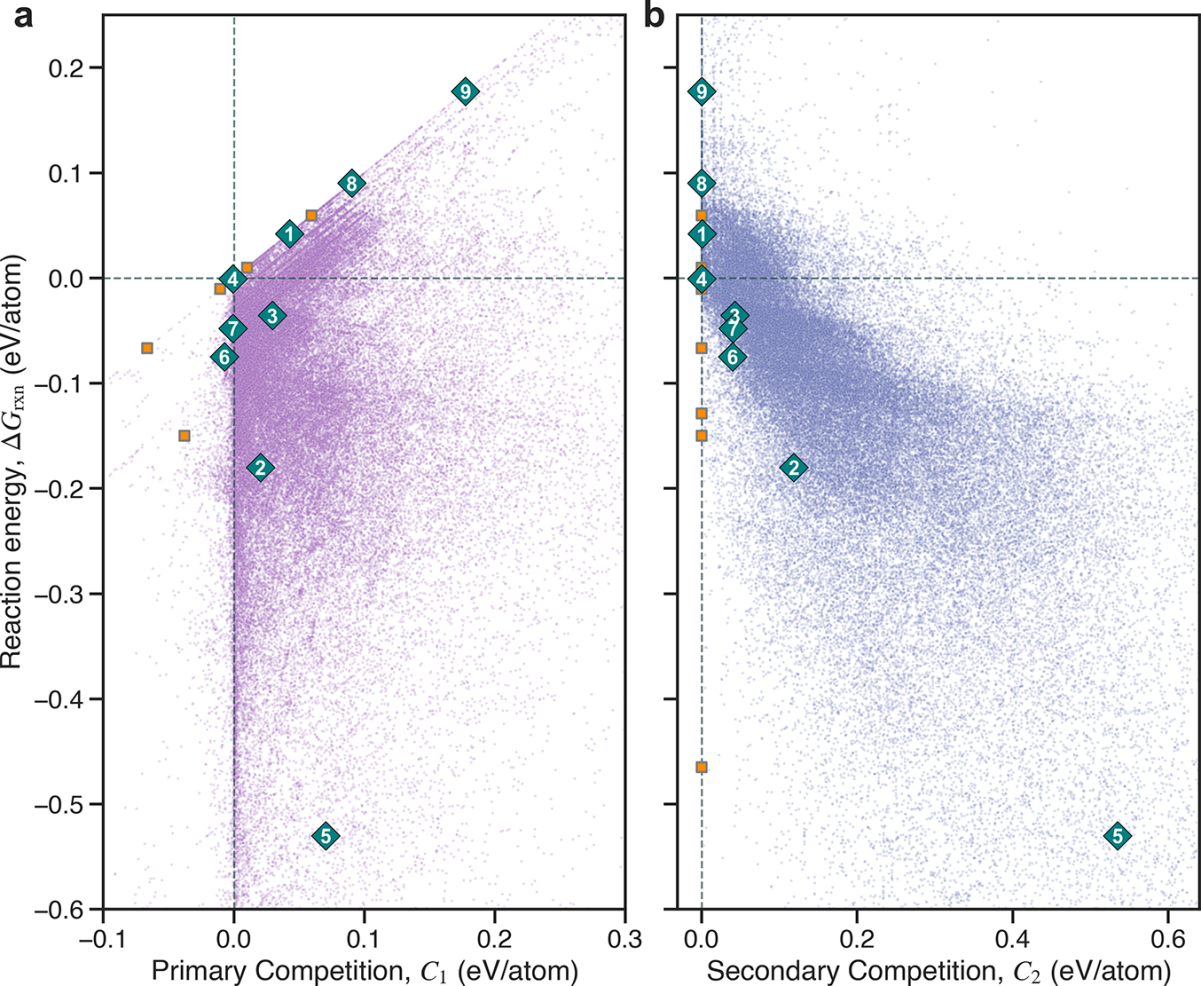
Synthesis maps of 82985 calculated reactions producing BaTiO_3_. Target reactions and their competition scores are extracted from an 18-element network of 2536160 reactions modeled at a temperature of *T* = 600 °C. Reactions are plotted on a shared axis of reaction energy, Δ*G*_rxn_, and on independent axes of (a) primary competition, *C*_1_, and (b) secondary competition, *C*_2_. The sharp boundaries are lower-bound limits of *C*_1_ = Δ*G*_rxn_ and *C*_2_ = 0. Blue diamonds indicate selected reactions experimentally tested in this work. Orange squares represent reactions on the three-dimensional Pareto front of Δ*G*_rxn_, *C*_1_, and *C*_2_.

Using our synthesis planning workflow, we computed and ranked 82985 binary (two-precursor) synthesis reactions producing BaTiO_3_, selected from a massive 18-element reaction network composed of all 2536160 enumerated binary reactions among 2417 phases with energies ≤0.050 meV/atom above the hull. To capture alternative chemistries (e.g., metathesis reactions, gas-forming reactions, ion exchange reactions, etc.), we selected a chemical system consisting of the target elements (Ba, Ti, O), alkali metals (Li, Na, K), alkaline-earth metals (Mg, Ca, Sr), halogens (F, Cl, Br), chalcogens (S), pnictogens (N, P), and other common elements (B, C, H). The Δ*G*_rxn_, *C*_1_, and *C*_2_ values for all calculated synthesis reactions are shown in the synthesis maps illustrated in Figure 5. We calculated these values at an intermediate temperature of *T* = 600 °C, which is near the median of our experimentally accessible temperature range (see Methods). We also considered the modeling of open-O_2_ reactions; similar results are shown in Figure S3 for 62133 open reactions in a more constrained subsystem.

Determining an optimal synthesis reaction can be formulated as an optimization problem of simultaneous minimization of the three reaction metrics: Δ*G*_rxn_, *C*_1_, and *C*_2_. A common approach for multiobjective optimization in synthesis planning is identifying the Pareto front.^14^ Here, we calculate a three-dimensional Pareto front for the BaTiO_3_ synthesis reactions (Table 3). The Pareto front reactions for the open-O_2_ system are provided in the Supporting Information.

**Table 3:** Pareto Front Reactions to BaTiO_3_ and Their Associated Gibbs Free Energies, Δ*G*_rxn_ (*T* = 600 °C), Primary Competition Scores, *C*_1_, Secondary Competition Scores, *C*_2_, and Costs, Γ^a^

rank	reaction	Δ*G*_rxn_	*C*_1_	*C*_2_	Γ	theoretical
1	2 TiP + 4.5 BaCO_3_ → BaTiO_3_ + Ba_3_(PO_4_)_2_ + 0.5 BaTi_2_O_5_ + 4.5 C	–0.465	–0.465	0.000	–0.256	
2	3 Ba(NO_3_)_2_ + 3.333 LiTi_2_N_3_ → BaTiO_3_ + 2 BaTi_2_O_5_ + 1.667 Li_2_TiO_3_ + 8 N_2_	–0.868	–0.302	0.000	–0.223	LiTi_2_N_3_
4	MgTiN_2_ + 0.6 Ba(NO_3_)_2_ → MgO + 0.2 BaTiO_3_ + 0.4 BaTi_2_O_5_ + 1.6 N_2_	–0.901	–0.223	0.008	–0.187	
6	8 O_2_ + Ba_4_TiP_4_ → BaTiO_3_ + Ba_3_P_4_O_13_	–2.330	0.062	0.136	–0.144	
7	4 BaO_2_ + 2 Ti → Ba_3_TiO_5_ + BaTiO_3_	–1.489	–0.021	0.036	–0.142	
18	0.25 Mg_4_TiN_4_ + 0.3 Ba(NO_3_)_2_ → MgO + 0.2 BaTiO_3_ + 0.05 Ba_2_TiO_4_ + 0.8 N_2_	–0.983	–0.057	0.078	–0.089	Mg_4_TiN_4_
28	TiO + BaO_2_ → BaTiO_3_	–1.044	–0.034	0.099	–0.075	
33	0.75 Ti_7_P_4_ + 0.3333 Ba_5_P_3_O_12_F → BaTiO_3_ + 0.25 Ba_2_TiO_4_ + 4 TiP + 0.1667 BaF_2_	–0.129	–0.129	0.000	–0.071	
54	MgTi(SO_4_)_3_ + 4 BaMg_2_ → BaTiO_3_ + 9 MgO + 3 BaS	–1.515	–0.005	0.225	–0.052	MgTi(SO_4_)_3_
63	4 Ti(NO_3_)_4_ + 5 Ba_4_P_2_O → Ba_2_TiO_4_ + 3 BaTiO_3_ + 5 Ba_3_(PO_4_)_2_ + 8 N_2_	–1.570	–0.018	0.261	–0.047	
85	2.25 TiC + 0.5 Ba_3_(PO_4_)_2_ → BaTiO_3_ + 0.25 Ba_2_TiO_4_ + TiP + 2.25 C	–0.067	–0.067	0.000	–0.037	
90	1.25 Ba_5_(TiN_3_)_2_ + 1.125 Ti(NO_3_)_4_ → BaTiO_3_ + 2.625 Ba_2_TiO_4_ + 6 N_2_	–1.287	–0.029	0.236	–0.035	
108	0.75 Ti_7_P_4_ + 0.5 Ba_3_(PO_4_)_2_ → BaTiO_3_ + 0.25 Ba_2_TiO_4_ + 4 TiP	–0.150	–0.038	0.000	–0.032	
199	1.45 Ti(ClO_4_)_4_ + 0.8 Ba_6_Mg_23_ → BaTiO_3_ + 0.45 Ba_2_TiO_4_ + 18.4 MgO + 2.9 BaCl_2_	–2.540	–0.008	0.527	–0.020	
259	3 BaO_2_ + Ti_2_N_2_O → BaTiO_3_ + Ba_2_TiO_4_ + N_2_	–0.913	–0.099	0.268	–0.015	Ti_2_N_2_O
498	TiNCl + 1.5 BaO_2_ → BaTiO_3_ + 0.5 BaCl_2_ + 0.5 N_2_	–0.967	–0.150	0.350	–0.007	
547	Li_2_TiO_3_ + 0.3333 Ba_3_(PO_4_)_2_ → BaTiO_3_ + 0.6667 Li_3_PO_4_	–0.011	–0.011	0.000	–0.006	
552	1.5 BaO_2_ + TiNF → BaTiO_3_ + 0.5 BaF_2_ + 0.5 N_2_	–0.960	–0.153	0.354	–0.006	TiNF
651	TiBrN + 1.5 BaO_2_ → BaTiO_3_ + 0.5 BaBr_2_ + 0.5 N_2_	–0.963	–0.152	0.357	–0.004	
1850	Ba_3_(PO_4_)_2_ + Ca_4_Ti_3_O_10_ → Ca_4_P_2_O_9_ + 3 BaTiO_3_	0.010	0.010	0.000	0.005	
2825	0.05882 Ba_2_Mg_17_ + 0.1118 Ti(NO_3_)_4_ → MgO + 0.1059 BaTiO_3_ + 0.005882 Ba_2_TiO_4_ + 0.2235 N_2_	–1.896	–0.004	0.447	0.010	
6598	3 Ti(SO_4_)_2_ + 8 BaMg_2_ → BaTiO_3_ + 16 MgO + 6 BaS + BaTi_2_O_5_	–1.628	–0.006	0.413	0.020	
7657	2 BaO_2_ + 0.25 NaTi_5_(NCl)_5_ → BaTiO_3_ + 0.25 Ba_2_TiO_4_ + 0.25 NaCl + 0.5 BaCl_2_ + 0.625 N_2_	–0.932	–0.079	0.336	0.023	NaTi_5_(NCl)_5_
8896	0.5 Ba_5_(TiN_3_)_2_ + 3 KNOF_2_ → BaTiO_3_ + 3 KF + 1.5 BaF_2_ + 3 N_2_	–1.170	–0.040	0.356	0.025	
13191	TiO_2_ + 0.5 Ba_2_Ca(BO_2_)_6_ → BaTiO_3_ + 0.5 Ca(B_3_O_5_)_2_	0.060	0.060	0.000	0.033	
15339	0.5 Ba_5_(TiN_3_)_2_ + 3 NOF → BaTiO_3_ + 1.5 BaF_2_ + 3 N_2_	–1.667	–0.058	0.509	0.036	
39153	0.5 Ba_5_(TiN_3_)_2_ + 1.5 SO_2_ → BaTiO_3_ + 1.5 BaS + 1.5 N_2_	–1.102	–0.042	0.450	0.074	
45245	0.5 Ti_2_S + 1.5 BaO_2_ → BaTiO_3_ + 0.5 BaS	–1.366	–0.068	0.563	0.086	
57844	0.8 Ba_5_(TiN_3_)_2_ + 0.45 Ti(ClO_4_)_4_ → BaTiO_3_ + 1.05 Ba_2_TiO_4_ + 0.9 BaCl_2_ + 2.4 N_2_	–1.729	–0.077	0.728	0.120	
64696	5 MgTiH_4_ + 3 Ba(NO_3_)_2_ → BaTiO_3_ + 5 MgO + 10 H_2_ + 2 BaTi_2_O_5_ + 3 N_2_	–1.142	–0.078	0.659	0.147	
65291	0.8 Ba_5_(TiN_3_)_2_ + 1.8 Br_2_O_3_ → BaTiO_3_ + 0.6 Ba_2_TiO_4_ + 1.8 BaBr_2_ + 2.4 N_2_	–1.810	–0.055	0.790	0.150	
65313	0.5 Ba_5_(TiN_3_)_2_ + 1.5 BrO_2_F → BaTiO_3_ + 1.5 BaBrF + 1.5 N_2_	–1.878	–0.053	0.804	0.150	
66253	3.5 Ba_5_(TiN_3_)_2_ + 9 ClO_3_ → BaTiO_3_ + 6 Ba_2_TiO_4_ + 4.5 BaCl_2_ + 10.5 N_2_	–1.841	–0.021	0.775	0.155	

a All units are in eV/atom.

Many Pareto-optimal reactions feature unconventional reactants and byproducts. BaO_2_ and Ba_5_(TiN_3_)_2_ are the most commonly appearing precursors (eight times each), followed by Ba(NO_3_)_2_ and Ba_3_(PO_4_)_2_ (four times each). Nearly all reactions (31 of 33) involve precursors containing additional elements other than Ba, Ti, and O. In particular, nitrogen is used in over half of the reactions (19 of 33), each featuring N_2_ gas formation. While unconventional, the high prevalence of nitride precursors in the Pareto front is not theoretically unreasonable; nitrides generally have less negative formation energies than oxides, making oxide formation with N_2_ gas evolution both energetically and entropically favorable.

However, many of the reactions appearing on the Pareto front are impractical from an experimental standpoint. For example, the aforementioned nitride precursors are likely challenging to synthesize and handle. Other Pareto-front reactions involve theoretical phases (e.g., LiTi_2_N_3_), uncommon or toxic precursors (e.g., Ba_5_(TiN_3_)_2_, SO_2_), difficult-to-remove byproducts (e.g., Ba_2_TiO_4_), or refractory precursors (e.g., TiC). Some of these suggested reactions can be removed easily with user-applied filters that account for specific experimental restrictions. For example, one can supply a list of available precursor compositions (i.e., “off-the-shelf” phases) or composition types to be avoided (e.g., sulfides, acids, etc.). In our provided code (see Methods), we support functionality for the former by including a list of hundreds of common precursors compiled from the catalogs of chemical suppliers. Filtering by these commonly available precursors (e.g., BaCO_3_, Ba_3_(PO_4_)_2_, TiO_2_) reduces the full set of 82985 BaTiO_3_ synthesis reactions to 478, making the generated recipes more easily parseable and readily testable. The filtered BaTiO_3_ reactions, including the corresponding open-O_2_ reactions, are provided in the Supporting Information. While filtering by conventional precursors is practically convenient, we consider the unorthodox nature of the *unfiltered* reactions an advantage of our approach, as this permits synthesis recommendations that expand beyond traditional chemical intuition. Still, synthesis recipes must be screened for reactivity, volatility, safety, and material costs. These challenges can be mitigated through the use of additional data or models; for example, reactivity can be approximated through surrogate data, such as defect formation energies or physical properties (melting points, hardness, etc.).^42^

We selected nine reactions from Figure 5 to test experimentally. The calculated thermodynamic metrics for these reactions are provided in Table 4. We intentionally selected reactions spanning various precursor chemistries, free energies, and competition scores. To prioritize the study of impurity-forming reactions and avoid the aforementioned practicality challenges, we did not explicitly include any reactions on the Pareto front. The conventional synthesis route, BaCO_3_ + TiO_2_ → BaTiO_3_ + CO_2_, was chosen as a baseline reference (Expt. 1). A positive energy is calculated for this reaction (Δ*G*_rxn_ = +0.042 eV/atom at *T* = 600 °C); however, this is likely due to residual uncorrected error in the calculated energy of BaCO_3_, which is not included in the NIST-JANAF data set. We did not test the analogous reaction with BaO precursor due to its hygroscopic nature, which makes it difficult to handle. However, we did test the alternative reaction from barium peroxide, BaO_2_ (Expt. 2). We included two reactions that form BaTiO_3_ directly from at least one other ternary phase (Expts. 3, 4). A reaction with a Ti metal precursor was selected due to its extremely high *C*_2_ score (Expt. 5). Two metathesis reactions (Expts. 6, 7) were selected due to their predicted high performance, including the unconventional use of a sulfide precursor (BaS). The final two reactions (Expts. 8, 9) were selected for being endergonic (Δ*G*_rxn_ > 0) to validate the accuracy of our free energy predictions. We note that several experiments feature precursors that are not easily purchasable from a chemical supplier (e.g., Ba_2_TiO_4_, Na_2_TiO_3_); these phases were synthesized following recipes reported in the literature (see Methods).

**Table 4:** Selected Experimental BaTiO_3_ Synthesis Reactions and Their Associated Gibbs Free Energies, Δ*G*_rxn_ (*T* = 600 °C), Primary Competition Scores, *C*_1_, Secondary Competition Scores, *C*_2_, and Costs, Γ

expt.	reaction	Δ*G*_rxn_ (eV/at)	*C*_1_ (eV/at)	*C*_2_ (eV/at)	Γ (eV/at)
1	BaCO_3_ + TiO_2_ → BaTiO_3_ + CO_2_	0.042	0.043	0.000	0.024
2	BaO_2_ + TiO_2_ → BaTiO_3_ + 0.5 O_2_	–0.180	0.021	0.119	0.045
3	Ba_2_TiO_4_ + TiO_2_ → 2 BaTiO_3_	–0.036	0.030	0.043	0.029
4	Ba_2_TiO_4_ + BaTi_2_O_5_ → 3 BaTiO_3_	–0.001	–0.001	0.000	–0.001
5	Ba(OH)_2_·H_2_O + 3.666 Ti → BaTiO_3_ + 1.333 Ti_2_H_3_	–0.530	0.070	0.534	0.219
6	BaCl_2_ + Na_2_TiO_3_ → BaTiO_3_ + 2 NaCl	–0.075	–0.007	0.040	0.007
7	BaS + Na_2_TiO_3_ → BaTiO_3_ + Na_2_S	–0.048	–0.001	0.041	0.013
8	2 BaS + 3 TiO_2_ → 2 BaTiO_3_ + TiS_2_	0.090	0.090	0.000	0.050
9	BaSO_4_ + 2 TiO_2_ → BaTiO_3_ + TiOSO_4_	0.178	0.178	0.000	0.098

**Figure 6:**
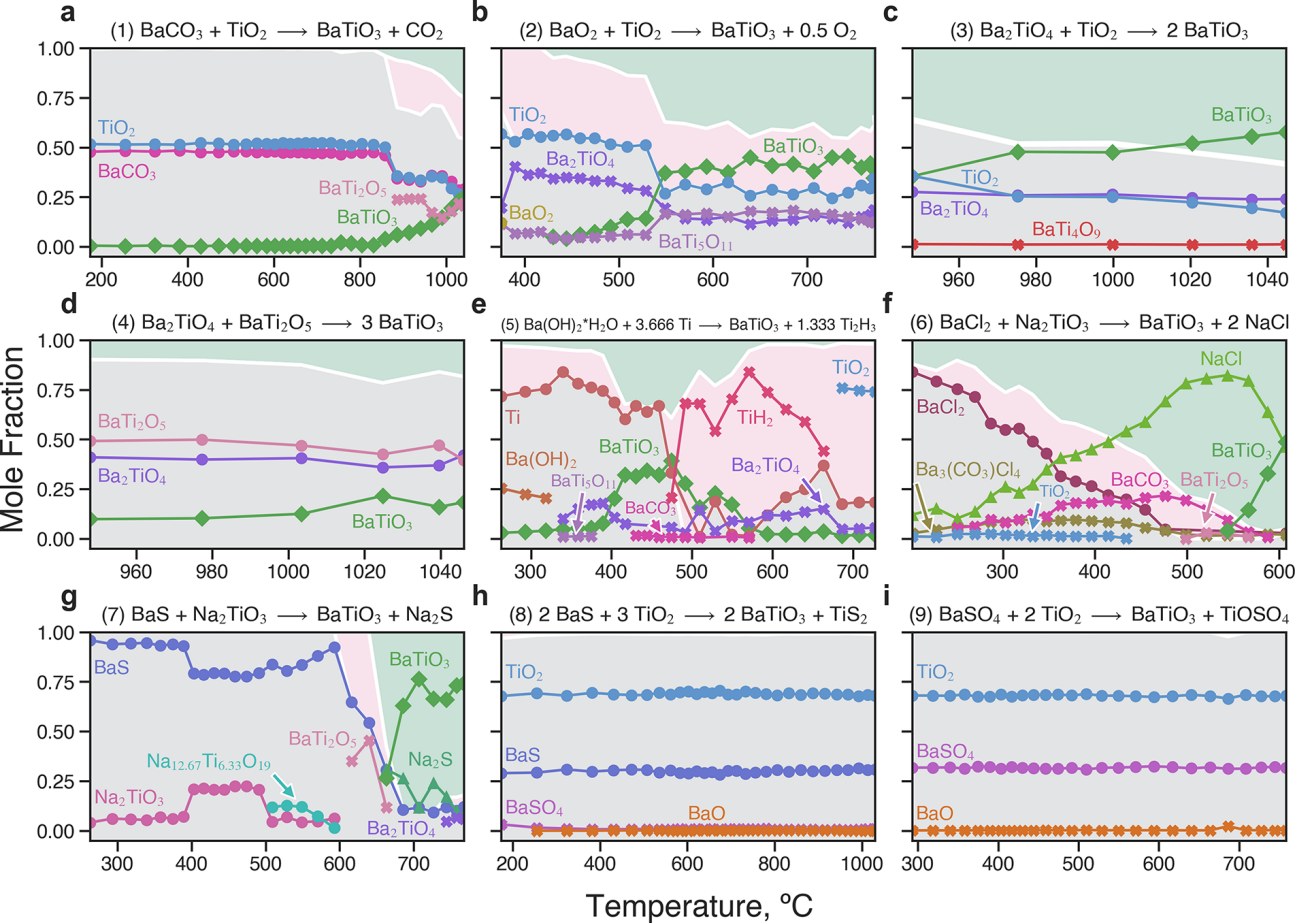
Reaction pathways for selected BaTiO_3_ synthesis experiments. (a–i) Mole fractions of observed phases for Expts. 1–9, respectively, as determined through Rietveld refinements of *ex post facto* SPXRD data. Phase types are distinguished by shape: precursors (circles), targets (diamonds), byproducts (triangles), and impurities (exes). Background shading denotes the total mole fractions of precursor (gray), impurity (pink), and target/byproduct (green). The median total reaction time was ∼67 min; exact times for each experiment are provided in Table S2.

The nine synthesis experiments were completed using a gradient furnace (see Methods),^43^ allowing the observation of reaction products over a wide range of temperatures (∼200–1000 °C) with *ex post facto* SPXRD. The experimental results are summarized in Figure 6. Mole fractions of each phase were determined via Rietveld refinement of SPXRD patterns acquired at various positions (temperatures) along the length of the sample after heating and subsequent cooling to ambient temperature. The *ex post facto* phase fraction plots can be interpreted similarly to those constructed with *in situ* data, as each effectively illustrates the reaction pathway during heating. More rigorously, however, each point in Figure 6 is pseudoindependent and best interpreted as the result of an isothermal (*ex situ*) reaction at its associated temperature. Shorter reaction times were selected to ensure capture of the onset of short-lived intermediate phases critical to assessing reaction pathway selectivities (Table S2). Selected Rietveld analysis results for each experiment are provided in Figures S4–S12 in the Supporting Information.

The observed reaction pathways demonstrate significant variation in target and impurity formation. Visualizing the interface reaction hulls of the selected experiments helps to rationalize their predicted and observed performance (Figure S13). The most complex of these pathways is that of Expt. 5, which features the formation of BaTiO_3_ at an intermediate temperature range (400–500 °C) before impurities Ba_2_TiO_4_, TiH_2_, and TiO_2_ begin to dominate. Indeed, Expt. 5 exhibits both the largest driving force (−0.530 eV/atom) and *C*_2_ value (0.534 eV/atom) of any reaction, supporting the observation of a complex reaction pathway containing many impurity phases.

The conventional synthesis reaction between BaCO_3_ and TiO_2_ (Expt. 1) was largely incomplete after 60 min at 1000 °C and exhibited significant formation of BaTi_2_O_5_. The interface reaction hull for this system (Figure S13a) suggests that the formation of BaTi_2_O_5_ is the most favorable reaction outcome, supporting our observations. In Expt. 2, the reaction of TiO_2_ with BaO_2_ appears to significantly decrease the reaction onset temperature but also features substantial impurity formation. In three of the experiments (Expts. 4, 6, and 7), the BaTiO_3_ synthesis reaction is the most favorable reaction on the hull, resulting in *C*_1_ < 0. Notably, the use of ternary precursor(s) in Expts. 3 and 4 (Ba_2_TiO_4_, BaTi_2_O_5_) results in low (or zero) *C*_2_, but also very little driving force. As a result, we observe near-perfect selectivity (i.e., very few visible impurities) at the expense of slowing down the reactions substantially. When the reaction energy is above zero, impurities associated with exergonic competing reactions may still form. For example, the hull for Expt. 8 indicates a significant degree of competition, including several reactions with only slightly positive energies (Δ*G*_rxn_ < 0.01 eV/atom). Expts. 8 and 9 are indeed largely unreacted as predicted but feature minor impurities (BaSO_4_ and/or BaO).

The metathesis reactions (Expts. 6, 7) show the overall greatest performance, yielding primarily BaTiO_3_ and the predicted byproducts at moderately low temperatures (600–700 °C). While metathesis reactions producing alkali halides are well-known for their optimal performance,^44^ it is a notable and surprising result that the sulfide-based reaction (BaS + Na_2_TiO_3_ → BaTiO_3_ + Na_2_S) achieves such pure and direct synthesis of BaTiO_3_, as predicted. Its success further highlights the importance of considering more complex chemistries involving additional elements besides those in the target phase (i.e., hyperdimensional chemistries).^40^ Some impurity formation, however, is evident in both metathesis reactions, particularly at lower temperatures. BaTi_2_O_5_ forms in both experiments and small amounts of Ba_2_TiO_4_ form in Expt. 7. However, this observation is supported by the calculated *C*_1_ and *C*_2_ scores, which indicate that neither reaction should be perfectly selective. Unexpectedly, the dominant impurities in Expt. 6 are carbonate compounds: Ba_3_(CO_3_)Cl_4_ and BaCO_3_. We presume this results from minor contamination of the precursors via reaction with CO_2_ in the air; some Na_2_CO_3_ observed in the precursor (see Methods) may have also contributed to the formation of the barium carbonate impurities via energetically favorable Ba/Na ion exchange reactions. For completeness, we have accounted for these unexpected impurities in our experimental analysis even though they were not explicitly considered in the selectivity calculations. However, we did exclude from consideration any Si-containing impurities such as Ba_2_TiSi_2_O_8_, which formed in small amounts due to reaction with the quartz capillaries; these Si impurities were minor (<2 mol %) and did not significantly affect our analysis.

**Figure 7:**
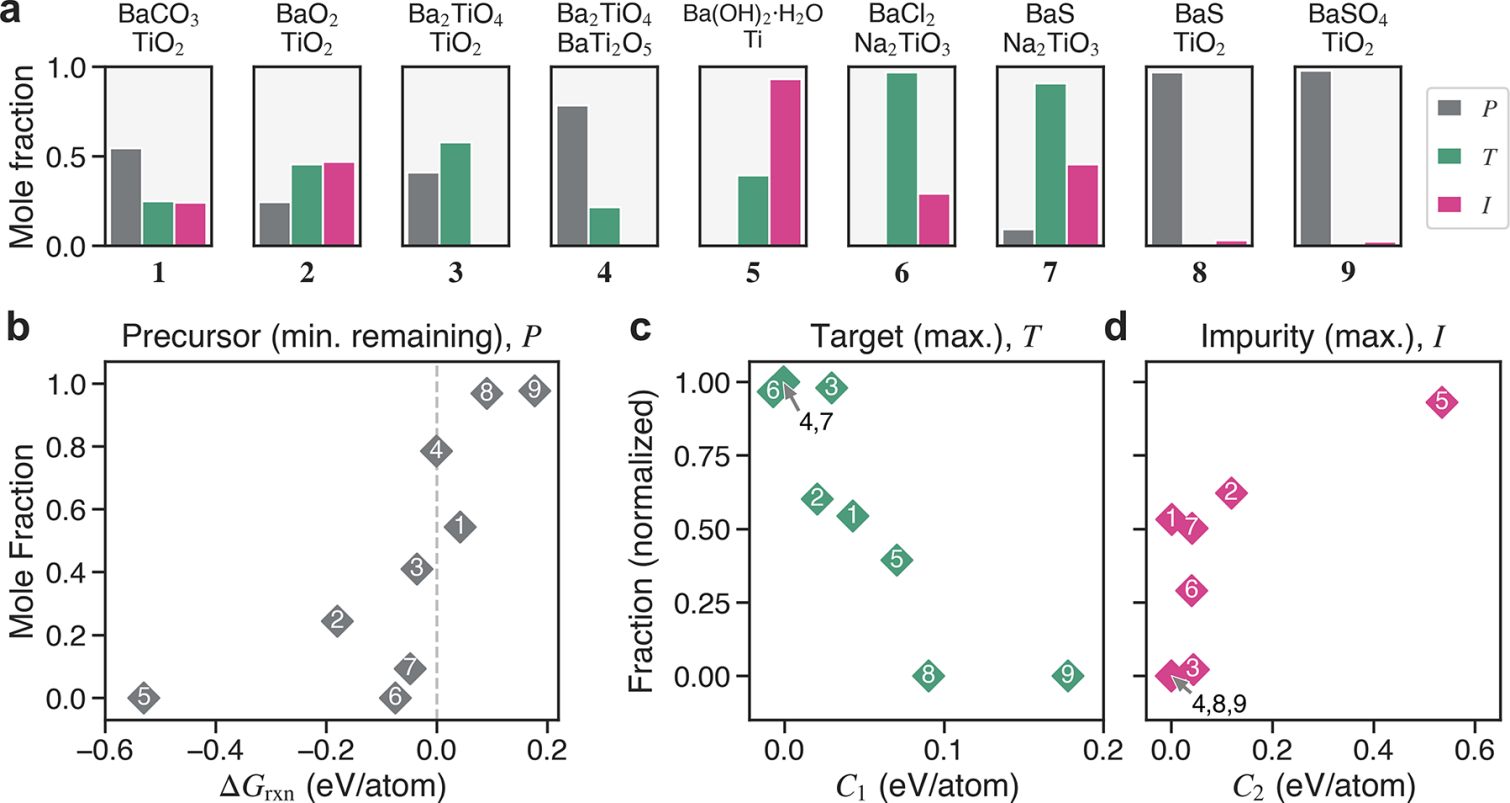
Summary of experimental results and correlations with calculated reaction metrics. (a) Precursor–Target–Impurity (PTI) plots, where the height of each bar indicates the relevant mole fraction captured at the most representative position (i.e., temperature) along the length of the sample after heating (and cooling). The different color bars correspond to the minimum amount of precursor remaining (*P*, gray), the maximum amount of target/byproducts formed (*T*, green), and the maximum amount of impurity formed (*I*, pink). (b) Positive correlation between *P* and the Gibbs free energy of the reaction, Δ*G*_rxn_. (c) Negative correlation between *T* and *C*_1_. (d) Positive correlation between *I* and *C*_2_. The *T* and *I* mole fractions have been normalized by the maximum amount of precursor consumed in the experiment, 1 – *P*, for enhanced visualization of the trend. The small impurity amounts detected in Expts. 8 and 9 are treated as yielding *I* = 0 to more accurately reflect the lack of observed reaction.

To quantitatively assess the performance of our predictions in determining the outcomes of experimental reactions, we propose three reaction outcome metrics summarizing the behavior of a reaction pathway: the minimum precursor remaining (*P*), the maximum target/byproduct formed (*T*), and the maximum impurity formed (*I*). Each metric is a mole fraction value taken from any data point (i.e., temperature) within the reaction pathway. This permits the capture of key features of the pathway independent of the kinetics of that reaction and is necessary given the range of chemistries explored. Our outcome metrics are visualized on the Precursor–Target–Impurity (PTI) plots in Figure 7a. The first quantity, *P* (minimum precursor remaining), gives insight into the reactivity and kinetics of the reaction: high values indicate reactions that did not complete at any temperature within the reaction time frame. The second quantity, *T* (maximum target/byproduct formed), provides a measure of the success of the reaction in producing BaTiO_3_ and predicted byproduct(s). Finally, the third quantity, *I* (maximum impurity formed), measures the selectivity of the reaction pathway, indicating the maximum fraction of intermediate/impurity phases synthesized at any temperature. Note that the temperature-independent PTI metrics are not required to sum to 1 for a particular experiment. This is intentional and advantageous because it permits the capture of poor selectivity in even nominally well-performing reactions; i.e., both *T* and *I* can be high (∼1) within the same experiment.

Figure 7b–d show selected correlations between reaction outcomes (*P*, *T*, and *I*) and the calculated reaction metrics (Δ*G*_rxn_, *C*_1_, and *C*_2_). The full set of all (3 × 3) pairwise correlation plots is available in Figure S14. We observe that the calculated reaction energy (Δ*G*_rxn_) correlates most strongly with the minimum amount of precursor remaining (*P*) at the conclusion of the experiment (Figure 7b). With infinite reaction times, we would theoretically expect this distribution to resemble a step function: *P* = 0 for reactions with Δ*G*_rxn_ < 0 and *P* = 1 for those with Δ*G*_rxn_ > 0. In our work, the distribution is less defined, given the shorter reaction times and different chemistries explored. The coordinates of Expts. 1 and 2 appear to deviate the most from a step-like distribution. Difficulty in modeling the energetics of carbonate reactions was previously discussed and likely explains the deviation of Expt. 1. In contrast, the deviation of Expt. 2 is likely kinetic in nature, as the reaction appears to stall (Figure 6b); this may suggest that the maximum temperature (750–800 °C) is too low to achieve sufficient reaction completion.

The primary (*C*_1_) and secondary (*C*_2_) competition metrics display negative and positive correlations with the maximum amounts of target (*T*) and impurity (*I*) phases formed, respectively (Figure 7c,d). We note that these trends are not strictly obeyed in a monotonic fashion. Still, the correlations are significant and can be theoretically rationalized by their derivation from the interface reaction hull. It is reasonable that *C*_1_ correlates most strongly with *T*, as this competition metric effectively measures the relative favorability of the target reaction over competing reactions. Similarly, it is sensible that *C*_2_ should correlate most strongly with *I*, given its derivation as a measure of the *relative* stability of impurity phases with respect to the target phase. To be precise, the experimental correlation between *C*_2_ and *I* suggests that one should consider not only the sum of the inverse hull distance energies for all competing phases but also the *total* energy of the entire secondary reaction sequence containing them (eq 2). By definition, this quantity includes, and thus will always be larger than or equal to, the sum of all competing inverse hull distance values for a particular interface reaction hull. Regarding the functional form of *C*_2_, the question arises as to whether a more simple summation of the *maximum* energy secondary reactions in the left and right hull subsections is a sufficient measure for secondary competition. While the maximum energy secondary reactions tend to account for much of the value of *C*_2_, on average, the full *C*_2_ metric is 0.077 eV/atom greater than that considering the maximum energy secondary reactions alone (Figure S15), suggesting that *C*_2_ is a more conservative metric. For completeness, we also tested an alternative formulation of the secondary competition metric using the enclosed “area” to the hull. While this metric correlates with *C*_2_, its calculation is more numerically unstable, and its units are less interpretable. Hence, we generally recommend the approach of modeling full secondary reaction sequences, which is straightforward to implement using our secondary competition algorithm (see Methods).

In a previous study,^24^ we suggested a solid-state reaction selectivity metric based on the difference in elemental chemical potentials between precursors and targets, measured by a distance along the chemical potential diagram (i.e., the “total chemical potential distance”). This metric was used to rationalize the unique selectivity of the Na-based precursor in synthesizing pyrochlore Y_2_Mn_2_O_7_ from YOCl and AMnO_2_ (A = Li, Na, K). While straightforward to compute using just the chemical potential diagram, the distance metric operates in the space of chemical potentials rather than reaction energies, rendering it less intuitive and more difficult to precisely discern the specific competing reactions. From the results here, we generally recommend that *C*_2_ be used instead of total chemical potential distance where possible. Both selectivity metrics capture similar characteristics of the competing phase space: each effectively involves the summation of competing phase stabilities through inverse hull distances or the corresponding chemical potential stability ranges. More precisely, both quantities are correlated (Figure S16) because chemical potentials are mathematical derivatives of the convex hull in energy-composition space. However, the total chemical potential distance is biased, particularly by competing phases with defective elemental-like compositions (e.g., Mg_149_Cl). Due to the increased weight of the entropic (−*TS*) term in the definition of Gibbs free energy, chemical potential diagrams featuring these compositions as competing phases may yield very high (unfavorable) total chemical potential distance values for synthesis reactions.

We acknowledge that while *C*_1_ and *C*_2_ are meant to capture different, independent mechanisms by which competing phases form, these metrics are at least *partially* correlated due to the geometric constraints of the convex hull. In particular, one situation is geometrically limited from occurring: high *C*_2_ and low *C*_1_ (Figure S17). Stated explicitly: if a competing phase lies significantly below the tie line formed by the target and a precursor (i.e., high *C*_2_), then both the target and that competing phase necessarily have similar reaction energies to form from the precursors, leading to high *C*_1_. In general, however, this restriction does not make the metrics redundant; while there is some correlation between the two, the correlation is not particularly strong (Figure S16). Therefore, we generally recommend the tandem use of both selectivity metrics.

The major limitation of our current synthesis planning workflow is the assumption that optimal synthesis reactions can be predicted with the thermodynamic energy landscape alone. While this is not the case for all chemistries, we show that, at least for chemical systems that exhibit practical solid-state reaction kinetics, the energy landscape alone can provide much of the rationalization for the observation of impurity phases. To say that impurity and secondary phases are inherently “kinetic” products is a misnomer. Rather, these phases may be the thermodynamic minima of smaller “local” interface systems, distinct from the thermodynamic products of the entire reaction mixture (the global thermodynamic solution). Furthermore, these impurities are often not easily convertible to final products without long-range mass transport or intervention (e.g., via regrinding or subsequent heating). This explains why impurities are often pervasive and challenging to remove in chemical systems with lower driving forces and/or slower kinetics (e.g., BiFeO_3_).

Although our current study focuses on the synthesis of oxides, we expect our synthesis planning approach to be suitable to other chemistries where solid-state synthesis can be employed. This includes the chemistries of most ionic compounds: halides, chalcogenides, pnictides, some silicides/carbides, etc. Still, one must ensure that there are enough thermodynamic data available to accurately model phase competition in the chemical system of interest. This is generally true for oxide compounds due to their high prevalence in literature and thermodynamic data; for example, currently, ∼53% of the nearly 150000 compounds in the Materials Project contain oxygen. While the predictive accuracy is currently greatest for oxides, we expect our approach to grow in accuracy and general applicability as computed materials databases grow in size and chemical complexity.

Currently, our workflow focuses exclusively on optimizing product purity; however, there are many issues one must consider when designing a synthesis recipe for a target compound: material cost, safety concerns, stability in air, handling challenges, availability of precursors, etc. Many routes suggested involve the formation of byproducts that are not easily removable from the product mixture (e.g., the formation of BaTiO_3_ with byproduct Ba_2_TiO_4_). To this point, one should be thoughtful in designing criteria by which to filter recommended synthetic routes. For example, one can prioritize the formation of only gaseous byproducts (e.g., O_2_ or CO_2_) or those easily removable by a solvent (e.g., NaCl). The cost function used to rank reactions can be modified to include other reaction metrics of interest, such as the estimated economic cost of the precursor materials. While not explicitly demonstrated here, the synthesis planning workflow can also be extended for application in multistep syntheses, allowing one to retrosynthetically sequence reactions to a target material beginning with purchasable, “off-the-shelf” precursors.

## Conclusions

Using the interface reaction model for powder reactions, we proposed two thermodynamic selectivity metrics for solid-state reactions: primary (*C*_1_) and secondary (*C*_2_) competition. To systematically and critically examine the effectiveness of our metrics, we analyzed existing successful synthesis routes available in the literature and, leveraging a massive set of 82985 synthesis reactions extracted from an 18-element reaction network constructed from Materials Project data, designed and executed nine BaTiO_3_ synthesis experiments with a range of selectivity values as compared to conventional precursors (BaCO_3_ and TiO_2_). Analysis of reaction pathways in the nine experiments via *ex post facto* synchrotron powder X-ray diffraction reveals that *C*_1_ and *C*_2_ correlate with the maximum amounts of target and impurity formed, respectively.

The main advantage of our approach compared to recent, existing approaches^14,25^ is the ability to simultaneously consider a wide range of chemistries, including those with unconventional additional elements. These so-called hyperdimensional chemistries^40^ allow one to bypass commonly encountered intermediates in target systems with many competing phases. This was demonstrated particularly for the BaTiO_3_ system studied in this work and is relevant for many other materials in the literature that are conventionally synthesized with theoretically suboptimal precursors (e.g., Na_2_Ti_3_O_7_, NaTaO_3_, LiMn_2_O_4_, etc.).

We anticipate that the selectivity metrics presented here and our computational synthesis planning workflow will significantly reduce the synthesis bottleneck, providing more rapid development of synthesis approaches for new, predicted materials. Our workflow provides a theoretical rationale for using certain precursors and synthesis conditions over other options, which promises to optimize existing synthesis procedures for current technologically important materials.

We envision our approach to be particularly useful in aiding high-throughput automated laboratory exploration efforts.^45^ Predictions can be used to design and downselect the synthesis reactions tested, reducing the cost and current trial-and-error approach to inorganic materials synthesis. The future inclusion of models for the kinetic behavior of reactions, such as estimates of the reactivity of precursors based on solid-state diffusivities, will further enhance predictions.

## Methods

### Thermodynamic Data

Gibbs free energies of formation, Δ*G*_f_(*T*), were acquired or approximated in a approach similar to those of previous works.^24,26^ We acquired experimental Δ*G*_f_(*T*) values from the NIST-JANAF thermochemical tables^29^ where available. Experimental values were limited to compounds with low melting points (i.e., *T*_m_ ≤ 1500 °C), as these systems demonstrate more complex phase change behavior over the temperature range studied here. For predominantly solid compounds (i.e., those with melting points above this threshold), as well as for all other phases not available in the NIST-JANAF thermochemical tables, we estimated Δ*G*_f_(*T*) using the machine-learned Gibbs free energy descriptor identified by Bartel et al.^28^ This descriptor was applied using formation enthalpies, Δ*H*_f_(*T* = 298 K), acquired from the Materials Project (MP) database,^27^ version 2022.10.28.

Due to the well-known and systematic formation energy error of carbonate compounds calculated with GGA exchange-correlation functionals,^14,19^ we applied an energy correction of 0.830 eV per CO_3_^2–^ anion to all carbonate compounds acquired from MP. This value was determined by fitting the mean error between computed and experimental Δ*G*_f_(*T* = 300 K) values for 15 metal carbonate compounds (Figure S18).

### Synthesis Planning Workflow

The synthesis reaction calculation and ranking procedure was implemented as a Python-based workflow in the existing *reaction-network* package.^26^ The code is available on GitHub at https://github.com/materialsproject/reaction-network. The workflow was constructed and launched on computing resources using the *jobflow*(46) and *fireworks*(47) workflow packages.

The synthesis planning workflow consists of three sequential steps. First, phases and their formation energies for the chemical system of interest are acquired as previously described. The total number of phases can be optionally reduced by setting a threshold for the maximum energy above hull (Δ*G*_hull_). In this work, we used a moderately large threshold of Δ*G*_hull_ ≤ 50 meV/atom, evaluated at ambient temperature (*T* = 300 K). Second, reaction enumeration is performed for the acquired phases using the combinatorial and free energy minimization approaches described in our previous work on solid-state reaction networks.^26^ Note that the combinatorial approach allows one to identify reaction product combinations above the hull (i.e., “metastable” products), which makes the analysis more robust to numerical error in the thermodynamic data. For systems with an open element (e.g., O_2_ gas), this reaction enumeration step is performed again using grand potential energies, where the open element has been assigned a user-defined value for the chemical potential (often the standard state, μ = μ^0^). Finally, *C*_1_ and *C*_2_ scores are calculated for all target synthesis reactions (i.e., those that form the desired target composition). To do this, the relevant competing reactions are extracted from the full set of enumerated reactions. We define a competing reaction as one whose precursors are a subset of the target reaction’s precursors. These competing reactions are then used to compute the interface reaction hull, from which *C*_1_ and *C*_2_ are calculated via eqs 1 and 4. For open systems, this selectivity calculation procedure is performed again, including any additional enumerated open reactions and ensuring that all reactions are calculated with grand potential energies at the corresponding chemical potential.

### Secondary Competition Algorithm

The secondary competition score, *C*_2_, is defined as the negative sum of the mean secondary reaction sequence energies to the left and right of the target on the interface reaction hull (eq 4). One approach for acquiring these quantities involves using a recursive algorithm to identify all possible sequences and their energies. However, this strategy is too slow for the high-throughput calculation of *C*_2_ in systems with many competing reactions.

Instead, we have identified a nonrecursive algorithm that takes advantage of the connection between this problem and the recursive construction of binary trees via the use of the Catalan number sequence. Our algorithm reformulates the sum of all secondary reaction sequence energies as a sum of individual secondary reaction energies weighted by their multiplicities, i.e., the total number of appearances of a particular reaction within the set of all possible secondary reaction sequences. The energy of any reaction indexed *k* can be calculated geometrically as the altitude, *h*_*k*_, of the triangle formed by its product vertex and two reactant vertices on the interface reaction hull. We find that the altitude multiplicity, *m*_*h*_*k*__, is determined to be the product of three Catalan numbers, *u*_*n*_, such that
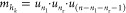
6where *n*_l_ and *n*_r_ refer to the number of interior vertices (i.e., within the triangle) to the left and right of the vertex of interest, respectively, and *n* is the total number of interior vertices for the entire hull subsection. For example, for secondary reactions between nearest neighbors, *n*_*l*_ = 0 and *n*_*r*_ = 0, resulting in an altitude multiplicity of *m*_*h*_ = *u*_*n*–1_.

The mean secondary reaction sequence energy for the hull subsection can then be calculated as
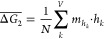
7where the sum occurs over all of the *V* unique reaction energies (altitudes), which is the number of unique triangles that can be constructed for the hull subsection, including the two exterior vertices: . The total number of unique secondary reaction sequences equals the corresponding Catalan number, *N* = *u*_*n*_. Finally, once this process has been performed for both the left and right hull subsections, the secondary competition (*C*_2_) can be calculated via eq 4.

### Literature Reactions

Solid-state literature reactions studied in this work were acquired from the text-mined data set of 31782 inorganic materials synthesis recipes originally extracted from the literature by Kononova et al.^33^ and available at https://github.com/CederGroupHub/text-mined-synthesis_public (version 2020-07-13). The original data set was filtered down to 8530 reactions that contain (1) precursors composed of ≤2 solids and ≤1 elemental gases (i.e., O_2_, H_2_, and N_2_), (2) no elements with an atomic number greater than 94 or for which the Gibbs free energy descriptor does not apply (e.g., Ne, Ar, Pm, Ra), (3) ten or fewer total elements due to limitations in the convex hull algorithm, and (4) no ions. Finally, these reactions were required to be stoichiometrically balanceable after adjusting compositions for hydrates and fractional formulas. For reactions containing variable compositions with one open variable (e.g., Nd_1–*x*_Sr_*x*_CoO_3_), we attempted to substitute all extracted values of *x* and retained the reactions that could be successfully balanced.

Competition metrics and free energies were assessed for each of the remaining reactions. For the enumerated competing reactions, metastable phases were considered up to a maximum threshold of Δ*G*_hull_ = 50 meV/atom, evaluated at ambient temperature (*T* = 300 K). Interface reaction hulls were constructed at the maximum temperature reported during synthesis, *T*_syn_. If this was not provided, a temperature of 800 °C was assumed. Formation energies, Δ*G*_f_(*T*_syn_), were assigned based on the ground-state energy for a given composition; i.e., we selected the lowest available formation energy of all polymorphs with the composition of interest. For increased accuracy, we did not include a reaction if any of its entries were missing from our thermodynamic data. For reactions with an open gas (i.e., O_2_, H_2_, N_2_), we assigned a chemical potential of μ_gas_ = 0 eV (i.e., standard state at *T*_syn_) for that element. For reactions completed in air, we assumed an O_2_ partial pressure of 0.21 atm and thus assigned a chemical potential of  eV. Finally, we removed duplicates with the same reaction equation and temperature/environment, as well as identity reactions (e.g., *A* → *A*). These filtering steps yielded a total of 3520 unique literature reactions.

### Precursor Materials

Precursors for all experiments were purchased from chemical providers or prepared via known solid-state synthesis approaches, as necessary. Precursors acquired from chemical providers include barium carbonate (BaCO_3_, J.T. Baker 99.9%), titanium(IV) oxide (anatase TiO_2_, Acros Organics 99.9%), barium sulfate (BaSO_4_, J.T. Baker 99.9%), barium hydroxide hydrate (Ba(OH)_2_·8H_2_O, Mathsen Colman & Bell 98%), barium chloride hydrate (BaCl_2_ ·2H_2_O, Fisher Scientific 99.9%), and titanium metal (Ti, annealed foil, Alfa Aesar 99.7%).

Precursors prepared via solid-state synthesis include barium orthotitante (Ba_2_TiO_4_), BaTi_2_O_5_, barium sulfide (BaS), and sodium metatitanate (Na_2_TiO_3_). Phase purities were assessed via laboratory powder X-ray diffraction (PXRD) analysis performed with a Bruker D8 Discover diffractometer using Cu Kα radiation.

Ba_2_TiO_4_ was prepared using stoichiometric amounts of BaCO_3_ and anatase TiO_2_.^48^ The chemicals were mixed, ground using a mortar and pestle, placed in an alumina boat inside of a mullite process tube with self-sealing end caps, and then heated at 950 °C for 16 h under Ar flow with a heating rate of 10 °C/min. The powder was then reground and reheated at 1100 °C for another 16 h at a heating rate of 10 °C/min. Handling operations were completed in an Ar glovebox due to the hygroscopic nature of Ba_2_TiO_4_. The product was phase-pure β-Ba_2_TiO_4_ with no observed impurities.

BaTi_2_O_5_ was prepared using stoichiometric amounts of BaCO_3_ and anatase TiO_2_.^49^ The chemicals were mixed, ground using a mortar and pestle, and heated in an alumina boat at 900 °C for 5 h as a pretreatment step. The powder was then reground and reheated at 1220–1225 °C for 24 h with heating and cooling steps of 3 h. The product was mostly phase pure with minor impurities, including a small amount of unreacted BaCO_3_ precursor (<3 mol %) and Ba_6_Ti_17_O_40_ (∼3 mol %). The latter phase was similarly observed in ref (49), where its formation was attributed to the thermodynamic instability of BaTi_2_O_5_ at temperatures outside a very narrow range (1220–1230 °C).

BaS was prepared using BaSO_4_ and activated carbon (C, J.T. Baker 99.9%).^50^ The chemicals were mixed, ground using a mortar and pestle, pressed into a 0.5 in. diameter pellet with 2 tons of force, and heated in an alumina boat at 1100 °C for 7–10 min in air, with a heating rate of 10 °C/min and natural cooling in the furnace. The product was phase pure with no detectable impurities.

Na_2_TiO_3_ was prepared using stoichiometric amounts of sodium hydroxide (NaOH, Fisher Scientific 99.9%) and anatase TiO_2_, with a slight excess of NaOH.^51^ The chemicals were mixed, ground using a mortar and pestle, and heated in an alumina boat at 500 °C for 2 h with a heating rate of 10 °C/min. The product was mostly phase pure with minor impurities. The sodium titanate peaks are best fit by a cubic α-Na_2_TiO_3_ structure with a small crystallite size. A minor amount of unreacted anatase TiO_2_ was present in the product (∼1 mol %). Na_2_CO_3_ also appears to be present as an impurity (∼11 mol %); we suspect this is due to contamination of the NaOH precursor via reaction with CO_2_ in the air.

### *Ex Post Facto* SPXRD Reactions

Synchrotron powder X-ray diffraction (SPXRD) data were collected in transmission (i.e., Debye–Scherrer) geometry on beamline 28-ID-2 (XPD) at the National Synchrotron Light Source-II (NSLS-II). Data were collected on a 2D area detector (PerkinElmer XRD 1621, 2048 × 2048 pixel array, 200 × 200 μm pixel size) at a sample-to-detector distance of 1407.1 mm using an incident X-ray energy of 68.12 keV (λ = 0.182 Å) with a 0.60 × 0.20 mm beam size. A total acquisition time of 1 s was used, summing five subframes collected for 0.2 s each.

Samples were packed into 1.1 mm OD/0.9 mm ID quartz capillaries. The capillary ends were filled with a 3 mm plug of powder silicon (Si, Strem 99.0%) followed by a cap of recycled silicon dioxide (SiO_2_). To account for possible gas production, the capillaries for Expts. 1, 2, 5, 7, and 8 were left unsealed, and a moderate vacuum (*P*_gage_ = −20 in Hg) was pulled on the samples during heating. All other sample capillaries (Expts. 3, 4, 6, and 9) were flame-sealed under argon.

Experiments were carried out in the gradient furnace described in ref (43), which heats samples to different temperatures across a range of spatial positions on the capillary (Figure S19). The furnace was operated with a Eurotherm 2408 temperature controller and a TDK Lambda 900W (30 V/30 A) power supply. Furnace heating elements were wound from resistive wire (Kanthal A-1, #24 awg). A K-type thermocouple (stainless steel, 0.01 in. OD) placed at an intermediate position along the sample was used as input for PID control of the furnace. We performed experiments in three temperature ranges with set points of *T*_H_ = 550 °C (Expts. 1, 3, 4, 8), *T*_L1_ = 450 °C (Expts. 2, 5, 6, 9), and *T*_L2_ = 400 °C (Expt. 7). This choice was motivated by differences in reactivity among the samples. Position-dependent temperatures were determined using a fit of measured *in situ* lattice expansion from NaCl/Si and Al_2_O_3_/MgO standards (Figure S20). Using the root-mean-square error of the curve fit, the estimated uncertainty for each temperature point is 11.4 °C (*T*_H_), 7.9 °C (*T*_L1_), and 10.9 °C (*T*_L2_). The experiments spanned a total temperature range of 189–1064 °C.

The median total time of each experiment was ∼67 min. Heating, holding, and cooling times varied among experiments due to differences in sample heat capacities, reactivities, and the sizes of investigated temperature windows; specifically, unreactive samples (Expts. 8, 9) and samples with smaller studied temperature windows (Expts. 3, 4) were held at elevated temperatures for shorter times. These differences are accounted for in our analysis via the use of relative metrics (i.e., mole fraction) and normalization by reaction progress. The exact heating, holding, and cooling times for each sample are shown in Table S2.

### Quantitative Phase Analysis of Powder Diffraction Data

Quantitative analysis of synchrotron powder X-ray powder diffraction data was carried out with the Rietveld method using either the TOPAS v6 (Expts. 1–3, 5, 7–9) or GSAS-II (Expts. 4, 6) software packages.^52,53^ Atomic displacement parameters were fixed to *B* = 1 Å^2^, and peak broadening was primarily modeled via crystal size broadening using a Lorentzian function. Site occupancies were fixed at 1.

## Supporting Information Available

Description of cost function power transformation, interface reaction hull for BaO|TiO_2_, open-O_2_ reaction data for BaTiO_3_, selected Rietveld refinements from all experiments, interface reaction hull plots for selected experimental reactions, selectivity metric correlations, details of carbonate correction, description of gradient furnace setup and temperature calibration, and table of experimental reaction times and temperature set points (PDF)
Table of 3520 reactions extracted from the literature with their calculated performance metrics and DOIs (XLSX)
Table of 82985 predicted/ranked closed BaTiO_3_ synthesis reactions (XLSX)
Table of 62133 predicted/ranked open-O_2_ BaTiO_3_ synthesis reactions (XLSX)
Table of 133 Pareto optimal open-O_2_ BaTiO_3_ synthesis reactions (XLSX)
Table of 478 predicted/ranked closed BaTiO_3_ synthesis reactions filtered by commonly available (i.e., purchasable) precursors (XLSX)
Table of 622 predicted/ranked open-O_2_ BaTiO_3_ synthesis reactions filtered by commonly available (i.e., purchasable) precursors (XLSX)
Transparent Peer Review report available (PDF)
