# Supplementary material for: Assessing Thermodynamic Selectivity of Solid-State Reactions for the Predictive Synthesis of Inorganic Materials
Source: ACS Cent Sci. 2023 Oct 16;9(10):1957–75. doi: 10.1021/acscentsci.3c01051 (PMC10604012; doi:10.1021/acscentsci.3c01051)
Supplement: Supplementary file 1 — oc3c01051_si_001.pdf [file oc3c01051_si_001.pdf]

Supporting Information:

Assessing Thermodynamic Selectivity of Solid-State Reactions for the Predictive Synthesis of Inorganic Materials

Matthew J. McDermott,[†,‡,@] Brennan C. McBride,[¶,@] Corlyn Regier,[¶] Gia Thinh Tran,[¶] Yu Chen,[†,‡] Adam A. Corrao,[§] Max C. Gallant,[†,‡] Gabrielle E. Kamm,[§] Christopher J. Bartel,[‖] Karena W. Chapman,[§] Peter G. Khalifah,[§,⊥] Gerbrand Ceder,[†,‡] James R. Neilson,[¶] and Kristin A. Persson[*,#,‡]

†*Materials Sciences Division, Lawrence Berkeley National Laboratory, Berkeley, CA*

‡*Department of Materials Science and Engineering, University of California, Berkeley, CA*

¶*Department of Chemistry, Colorado State University, Fort Collins, CO*

§*Department of Chemistry, Stony Brook University, Stony Brook, NY*

‖*Department of Chemical Engineering and Materials Science, University of Minnesota, Minneapolis, MN*

⊥*Chemistry Division, Brookhaven National Laboratory, Upton, NY*

#*Molecular Foundry, Lawrence Berkeley National Laboratory, Berkeley, CA*

@*These authors contributed equally to this work.*

E-mail: kapersson@lbl.gov

Power transform of costs in literature reactions

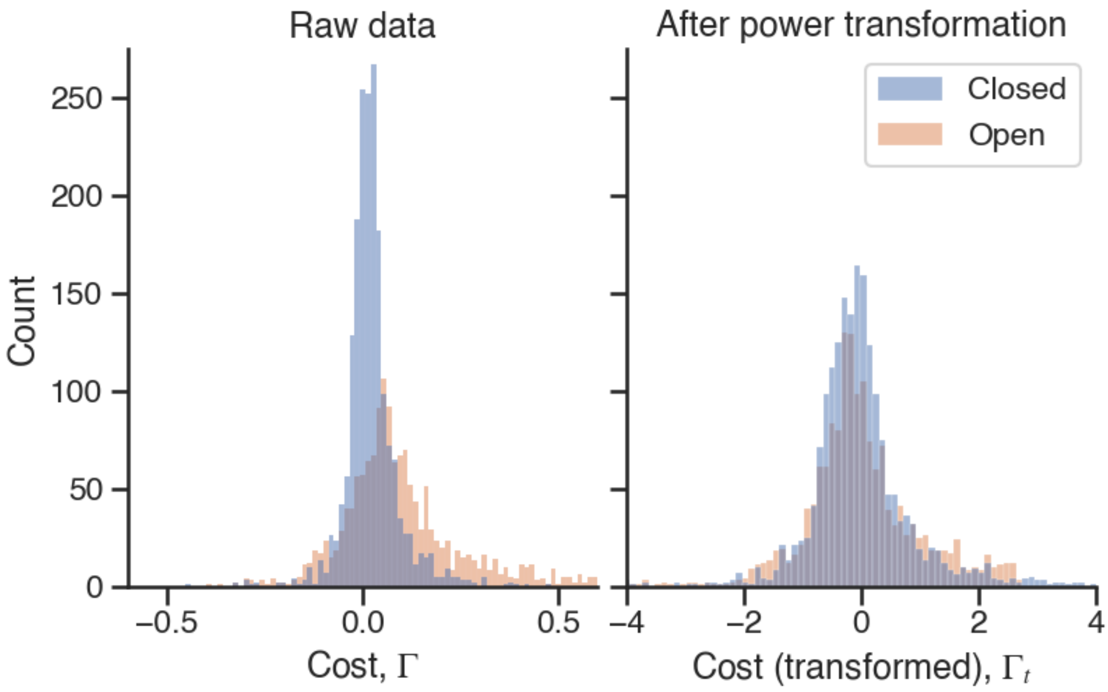

Figure S1: Power transformation applied to literature reaction costs, Γ. The power transform monotonically transforms the reaction costs for closed and open reactions such that they resemble standard normal distributions, allowing for better comparison between the two datasets.

BaO|TiO_2 interface reaction hull

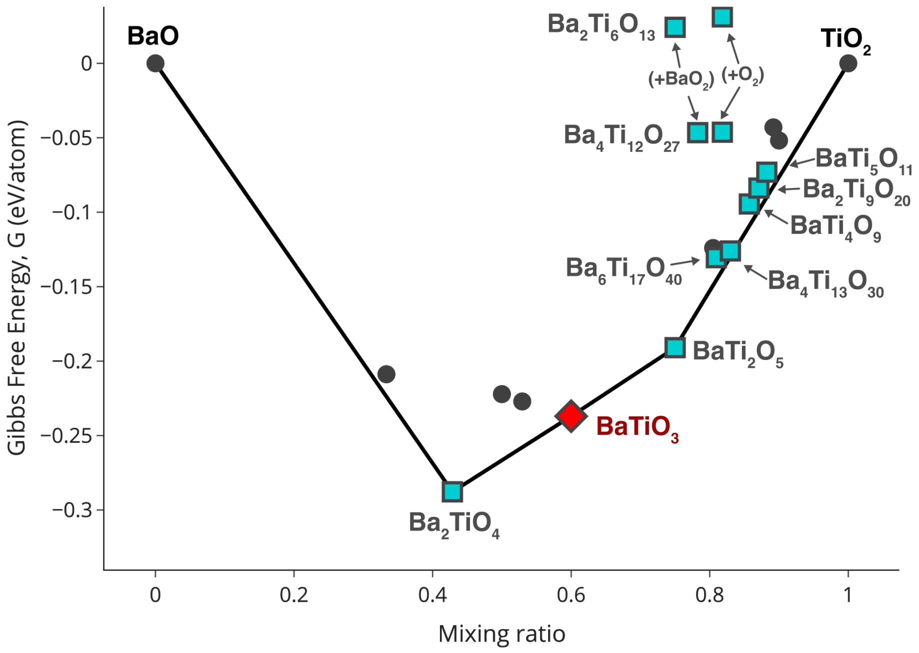

Figure S2: Calculated interface reaction hull for BaO|TiO_2 at T = 600 °C. Blue squares denote experimentally known ternary phases in the system. Ba_2TiO_4 is the most commonly observed intermediate/impurity in standard syntheses of $BaTiO_3$ from BaO (or $BaCO_3$) and TiO_2; it is also predicted to be the phase with the greatest driving force to form.

$BaTiO_3$ synthesis reactions: open O_2

Table S1: Selected experimental reactions to $BaTiO_3$ and their associated grand potential energies ($\mu_O = -0.1001$ eV), $\Delta\Phi_{\mathrm{rxn}}$ ($T = 600$ ℃), primary competition scores, C_1, secondary competition scores, C_2, and costs, Γ.

Expt.	Reaction	$\Delta\Phi_{\mathrm{rxn}}$ (eV/at)	C_1 (eV/at)	C_2 (eV/at)	Γ (eV/at)
**1**	$BaCO_3 + TiO_2 \longrightarrow BaTiO_3 + CO_2$	0.112	0.114	0.001	0.063
**2**	$BaO_2 + TiO_2 \longrightarrow BaTiO_3 + 0.5\ O_2$	-0.590	0.078	0.389	0.151
**3**	$Ba_2TiO_4 + TiO_2 \longrightarrow 2\ BaTiO_3$	-0.089	0.085	0.114	0.081
**4**	$Ba_2TiO_4 + BaTi_2O_5 \longrightarrow 3\ BaTiO_3$	-0.002	-0.002	0.000	-0.001
**5**	$Ba(OH)_2{\cdot}H_2O + 3.666\ Ti \longrightarrow BaTiO_3 + 1.333\ Ti_2H_3$	-0.713	8.188	8.973	7.651
**6**	$BaCl_2 + Na_2TiO_3 \longrightarrow BaTiO_3 + 2\ NaCl$	-0.112	-0.004	0.077	0.021
**7**	$BaS + Na_2TiO_3 \longrightarrow BaTiO_3 + Na_2S$	-0.077	4.032	4.174	3.685
**8**	$2\ BaS + 3\ TiO_2 \longrightarrow 2\ BaTiO_3 + TiS_2$	0.168	4.276	4.109	3.790
**9**	$BaSO_4 + 2\ TiO_2 \longrightarrow BaTiO_3 + TiOSO_4$	0.533	0.533	-0.000	0.293

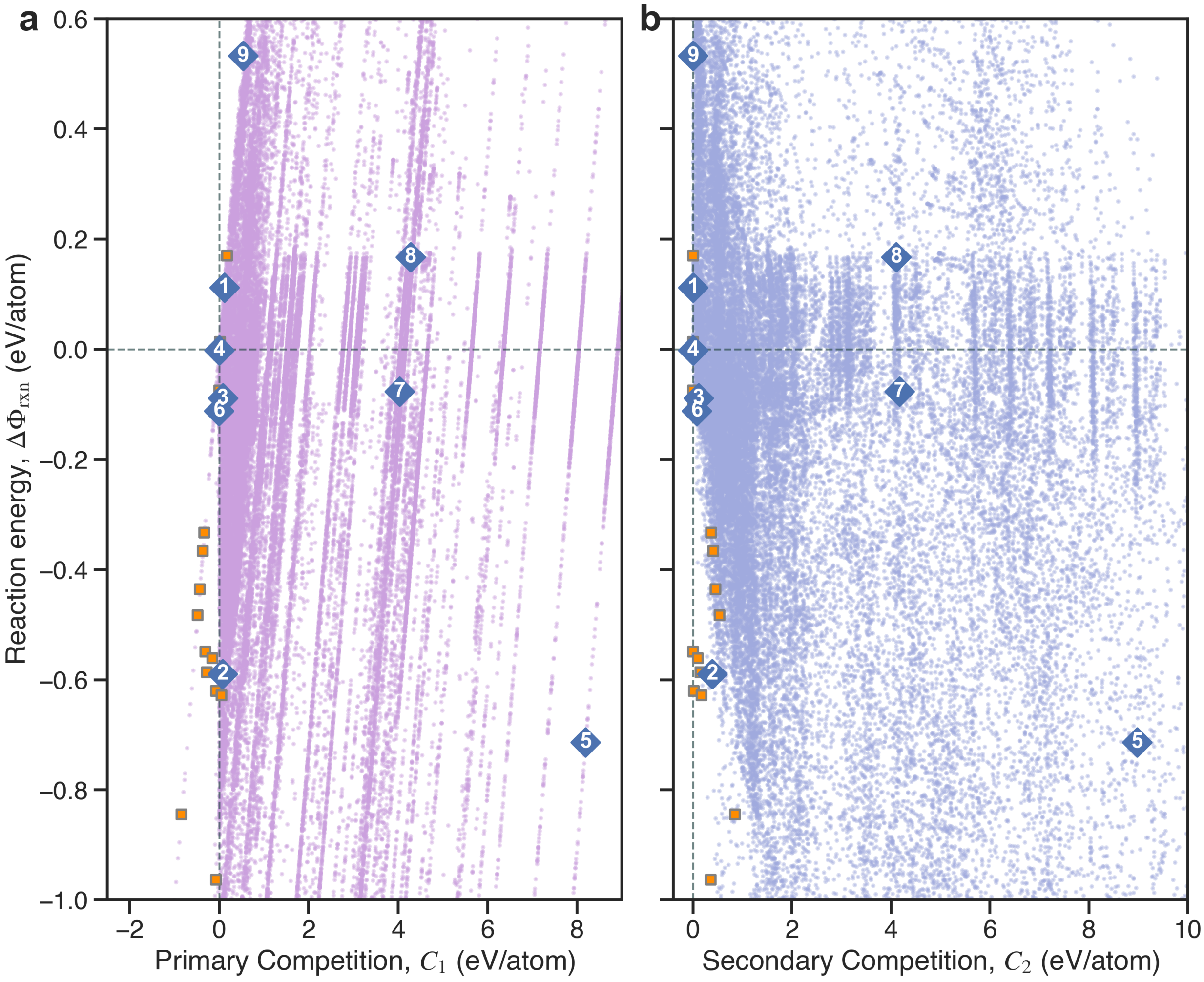

Figure S3: Synthesis map of 62,133 calculated open-O_2 synthesis reactions producing $BaTiO_3$. Reaction energies and competition scores are calculated assuming a temperature of $T = 600$ ℃ and oxygen chemical potential $\mu_{\mathrm{O}} = -0.1001$ eV, as approximated by selected experimental conditions (vacuum at $P_{\mathrm{gage}} = -20$ in Hg). As in Figure 5, reactions are plotted on a shared axis of reaction energy, $\Delta\Phi_{\mathrm{rxn}}$, and on independent axes of (a) primary competition, C_1, and (b) secondary competition, C_2. Orange squares represent reactions on the three-dimensional Pareto frontier of $\Delta\Phi_{\mathrm{rxn}}$, C_1, and C_2. Blue diamonds indicate selected reactions experimentally tested in this work.

Selected Rietveld refinements for $BaTiO_3$ experiments

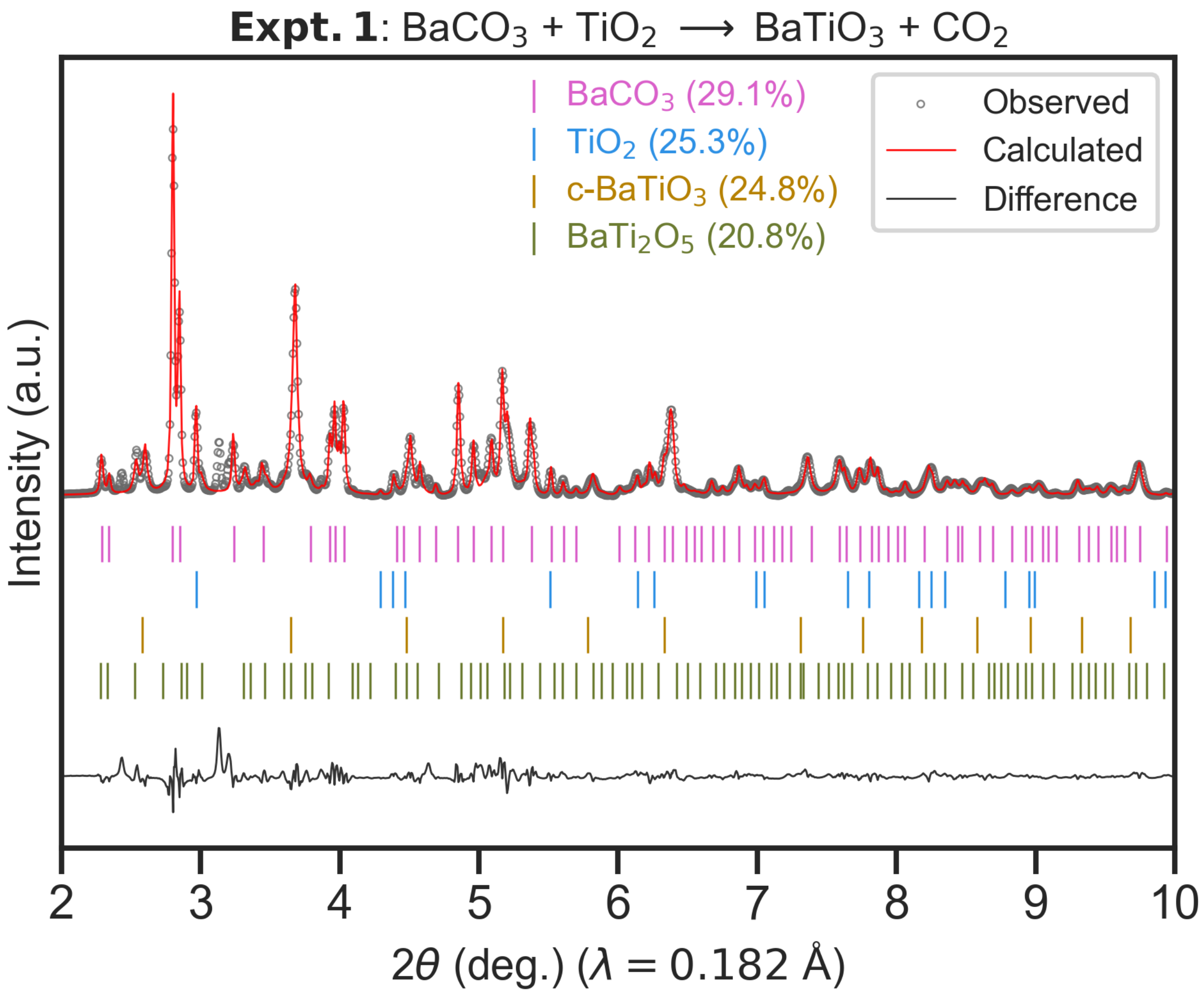

Figure S4: Selected Rietveld refinement for Experiment 1. The observed pattern represents *ex post facto* synchrotron powder X-ray diffraction data (SPXRD) captured following reaction at T = 1040 °C, which corresponds to the temperature with the highest $BaTiO_3$ yield. Phase fractions are shown in units of mole percent.

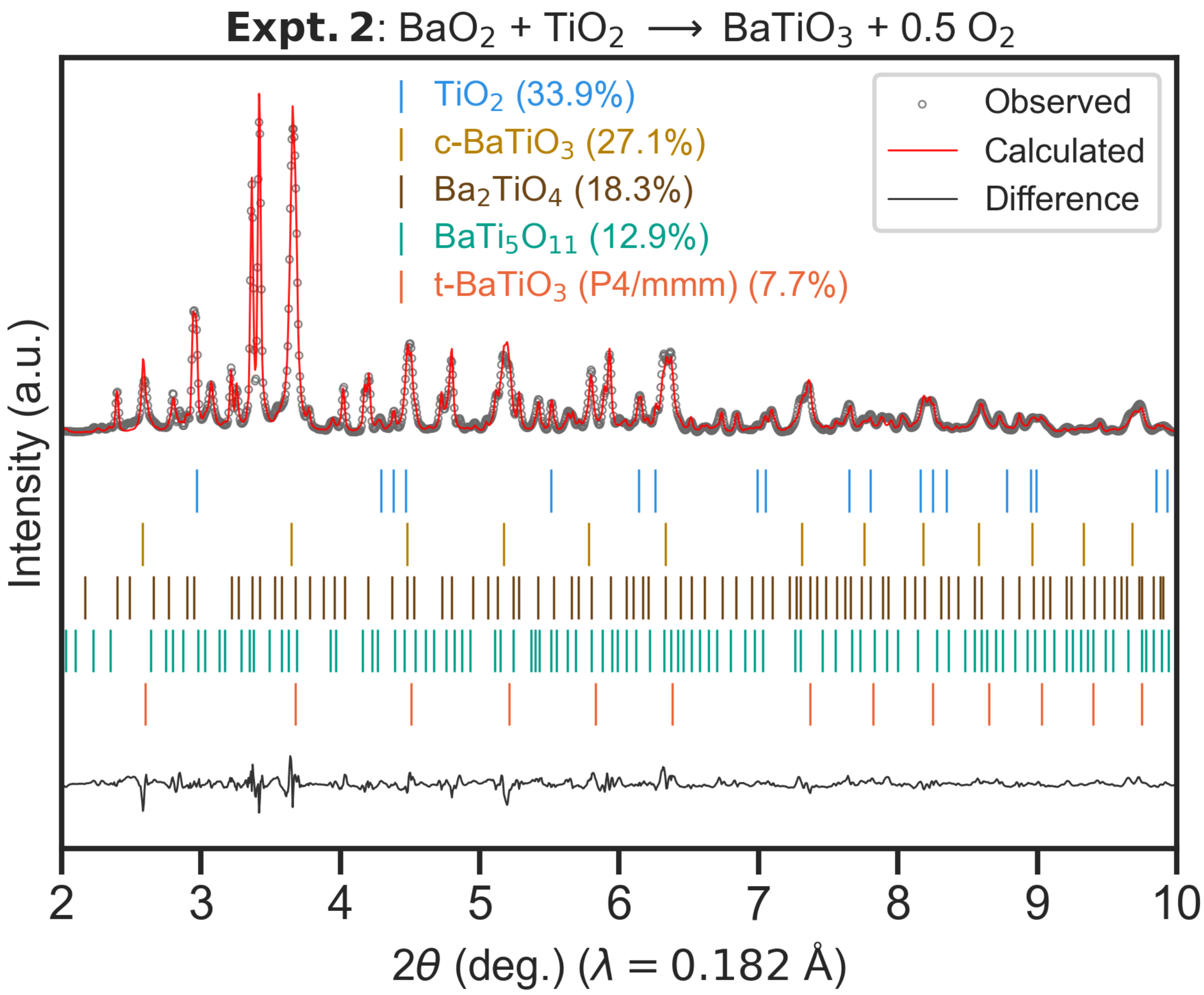

Figure S5: Selected Rietveld refinement for Experiment 2. The observed pattern represents *ex post facto* SPXRD data captured following reaction at T = 762 ℃, which corresponds to the temperature with the highest $BaTiO_3$ yield. Phase fractions are shown in units of mole percent.

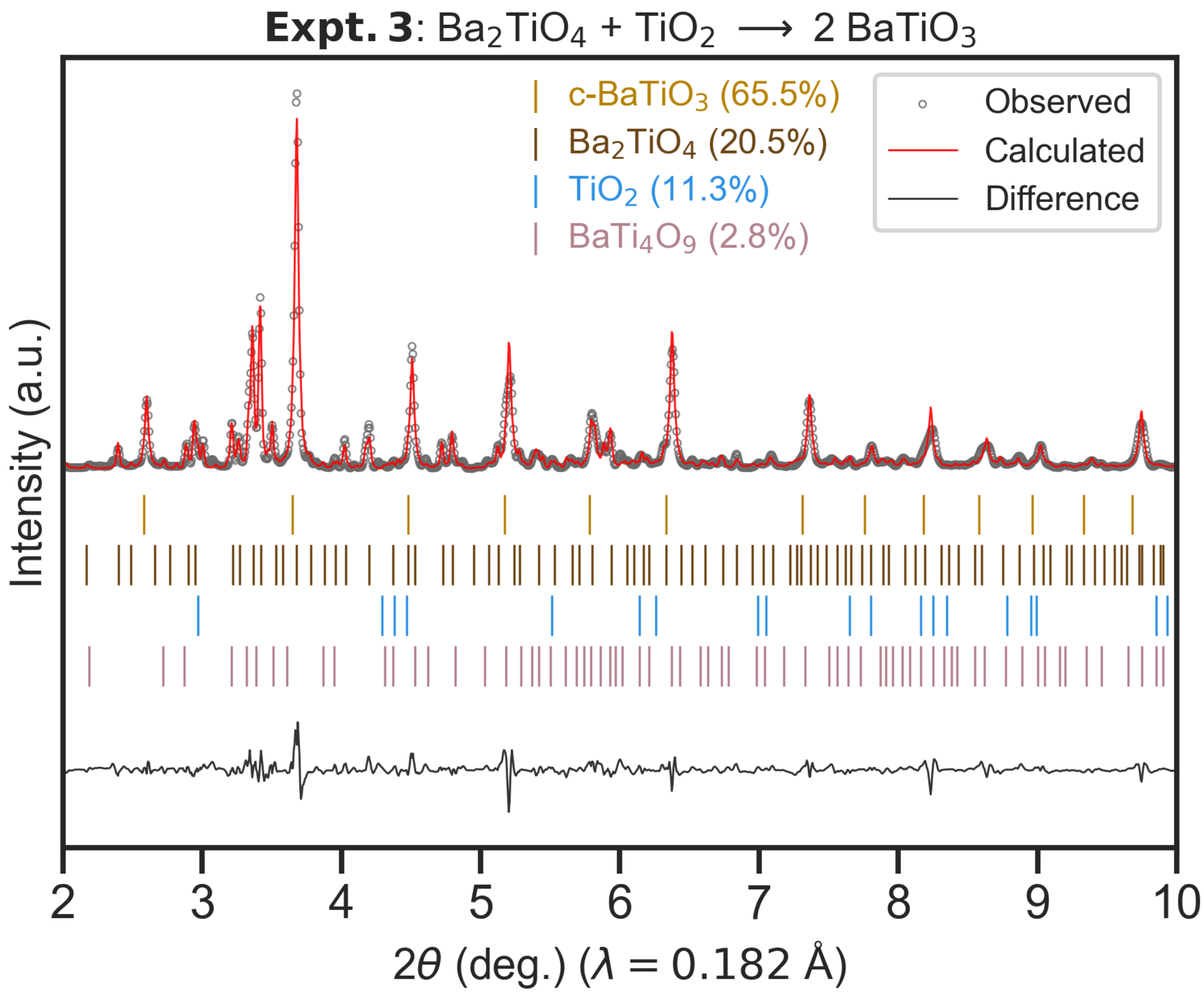

Figure S6: Selected Rietveld refinement for Experiment 3. The observed pattern represents *ex post facto* SPXRD data captured following reaction at T = 1045 ℃, which corresponds to the temperature with the highest $BaTiO_3$ yield. Phase fractions are shown in units of mole percent.

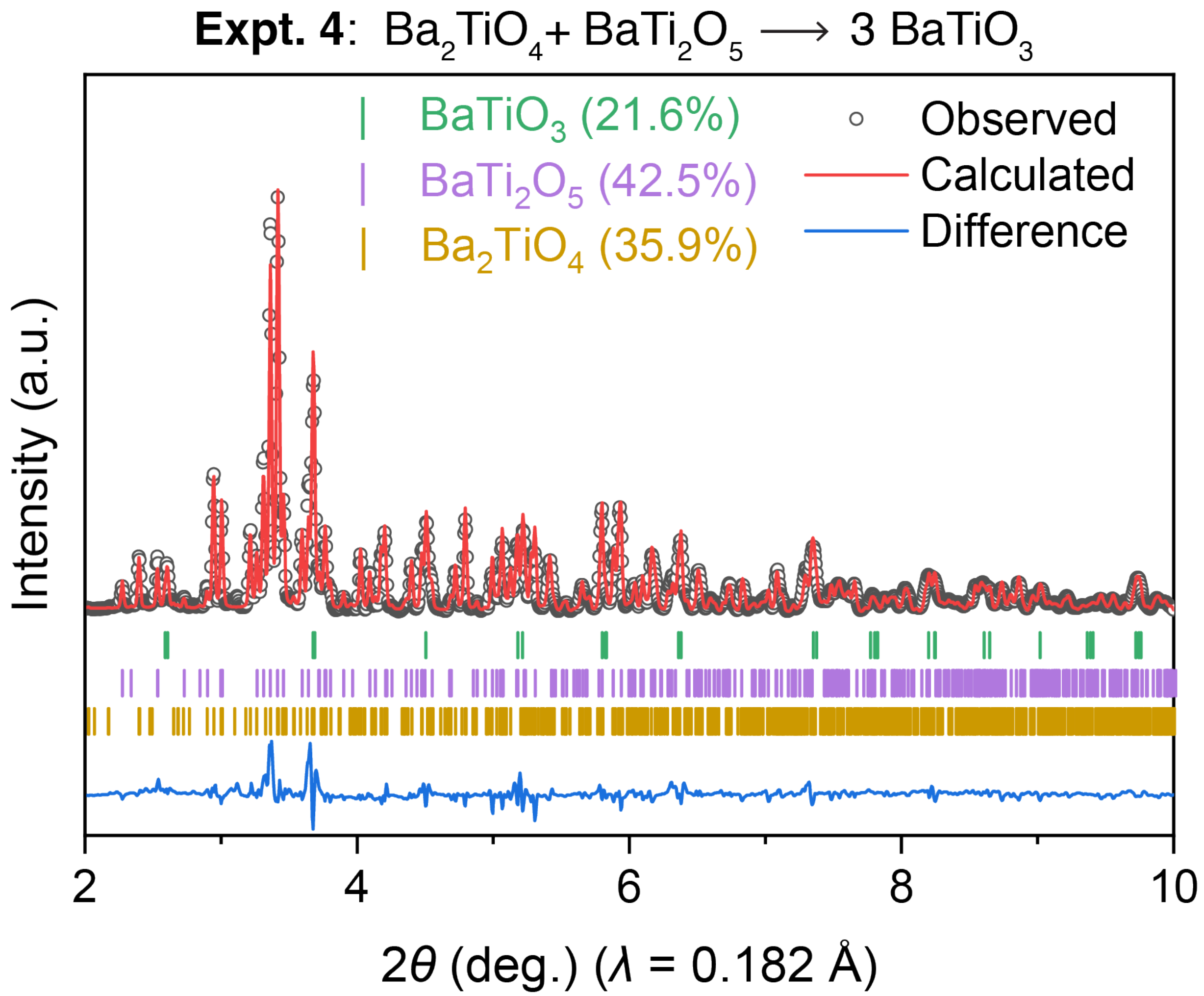

Figure S7: Selected Rietveld refinement for Experiment 4. The observed pattern represents *ex post facto* SPXRD data captured following reaction at T = 1025 ℃, which corresponds to the temperature with the highest $BaTiO_3$ yield. Phase fractions are shown in units of mole percent.

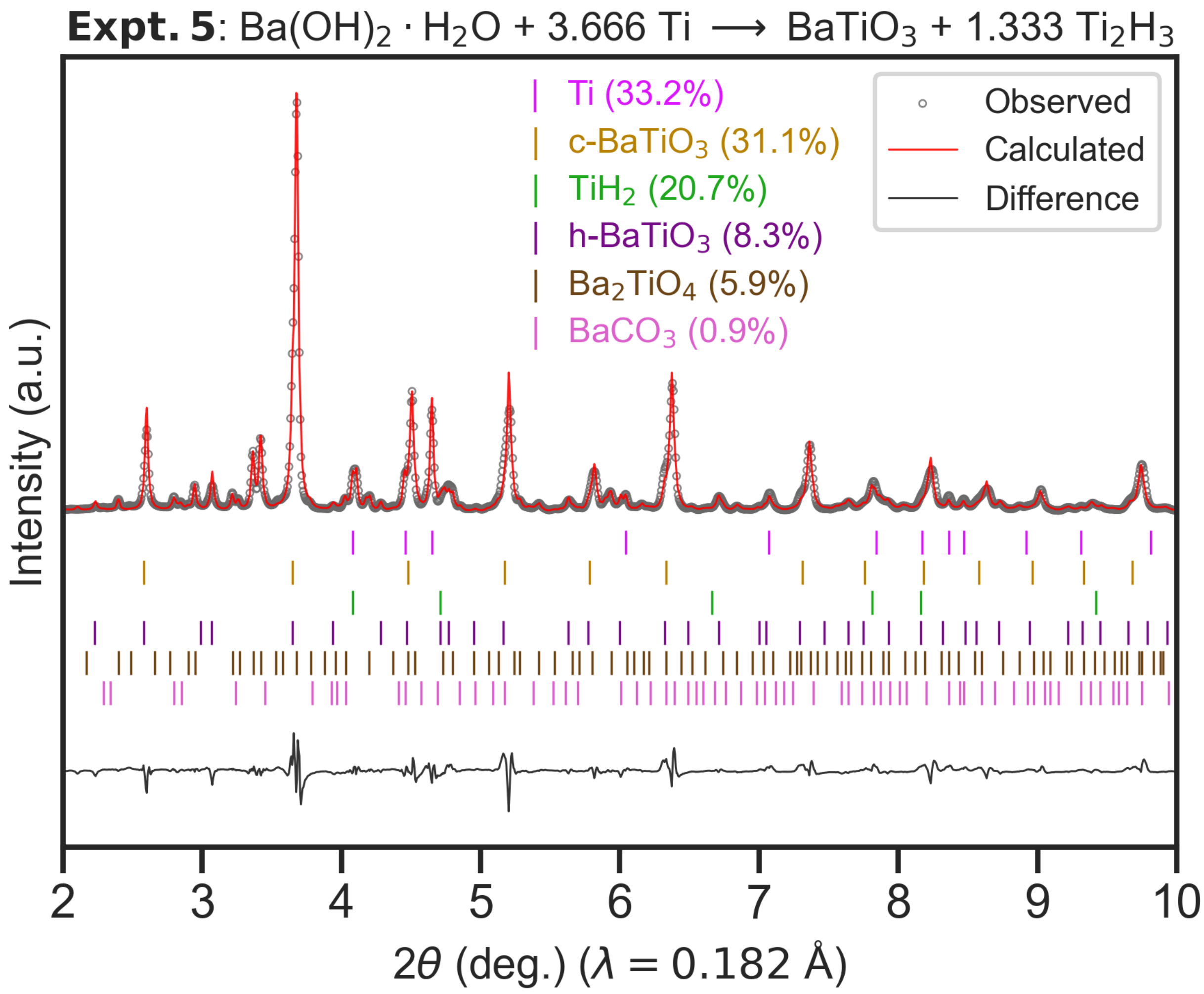

Figure S8: Selected Rietveld refinement for Experiment 5. The observed pattern represents *ex post facto* SPXRD data captured following reaction at T = 474 ℃, which corresponds to the temperature with the highest $BaTiO_3$ yield. Phase fractions are shown in units of mole percent.

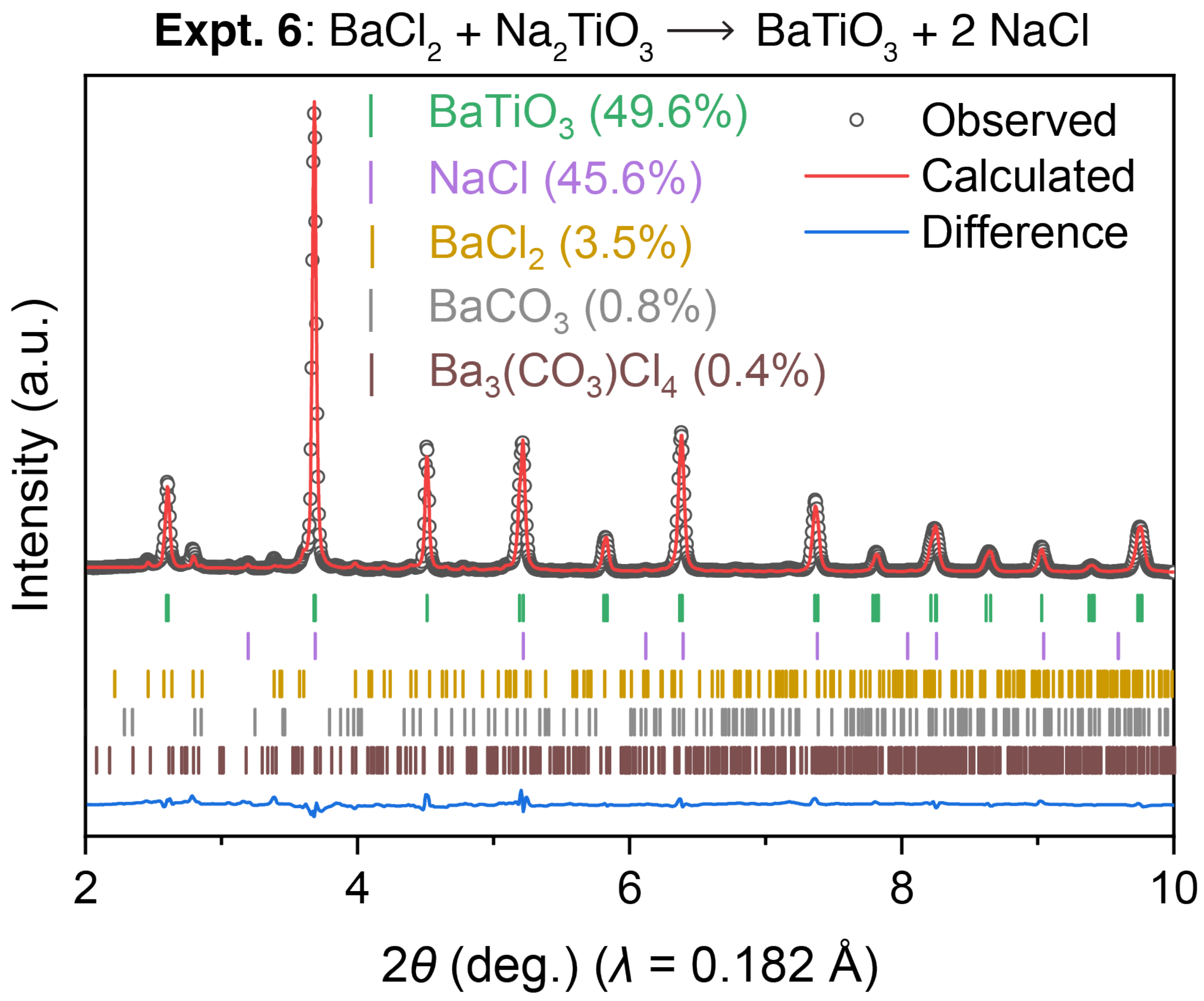

Figure S9: Selected Rietveld refinement for Experiment 6. The observed pattern represents *ex post facto* SPXRD data captured following reaction at T = 625 ℃, which corresponds to the temperature with the highest $BaTiO_3$ yield. Phase fractions are shown in units of mole percent.

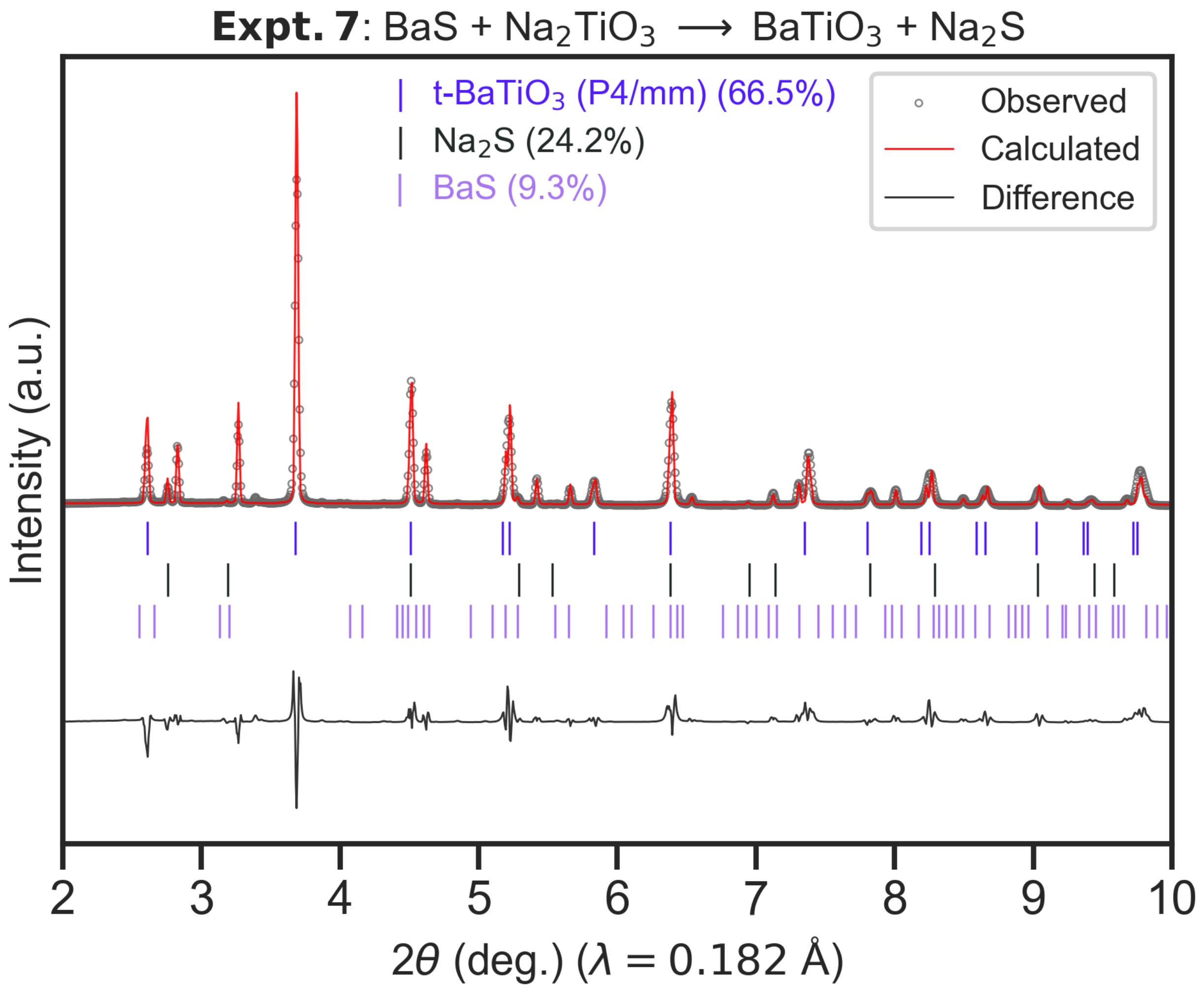

Figure S10: Selected Rietveld refinement for Experiment 7. The observed pattern represents *ex post facto* SPXRD data captured following reaction at T = 727 ℃, which corresponds to the temperature with the highest $BaTiO_3$ yield. Phase fractions are shown in units of mole percent.

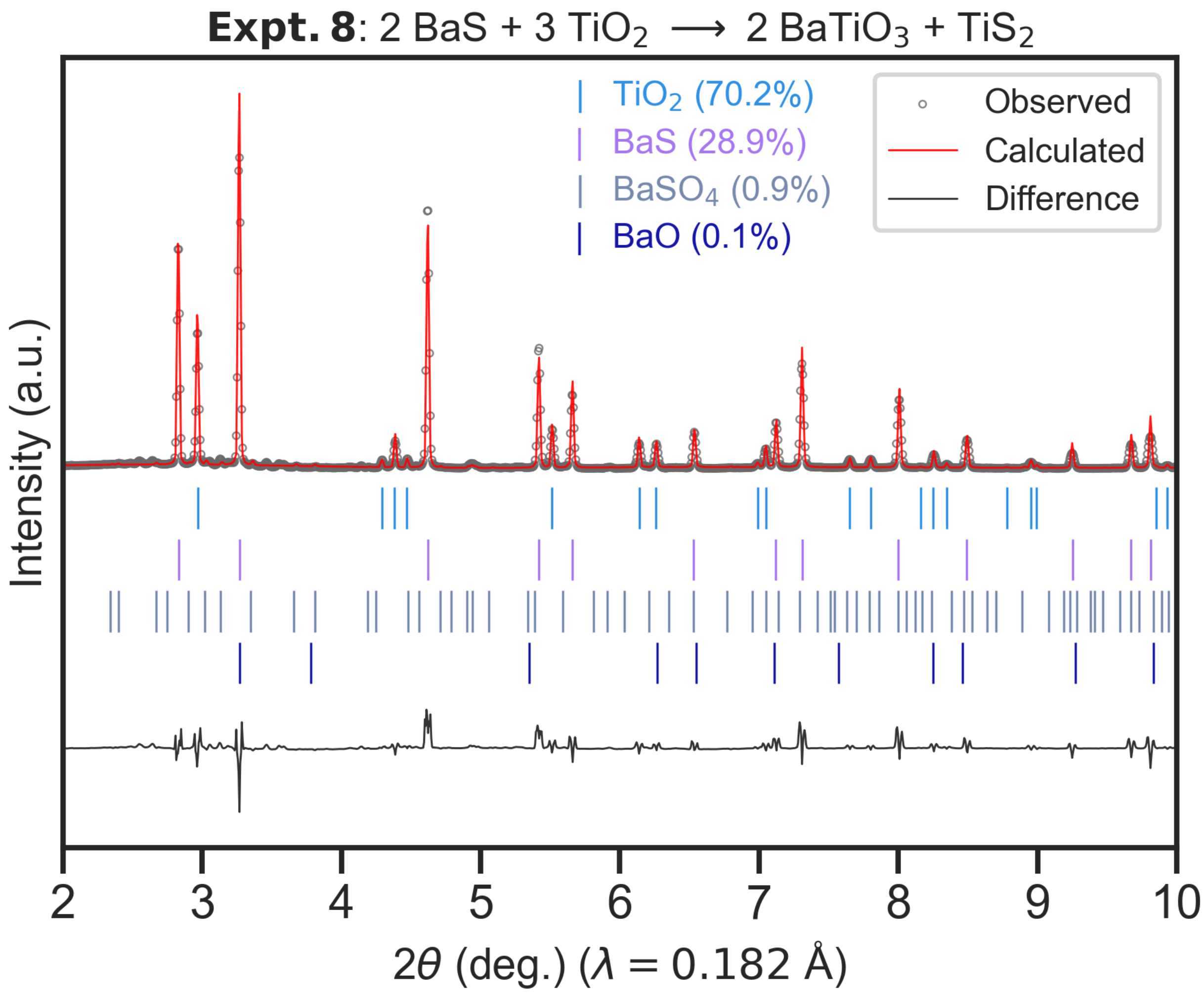

Figure S11: Selected Rietveld refinement for Experiment 8. The observed pattern represents *ex post facto* SPXRD data captured following reaction at T = 603 °C. This value corresponds to the median temperature studied since $BaTiO_3$ was not observed at any temperature. Phase fractions are shown in units of mole percent.

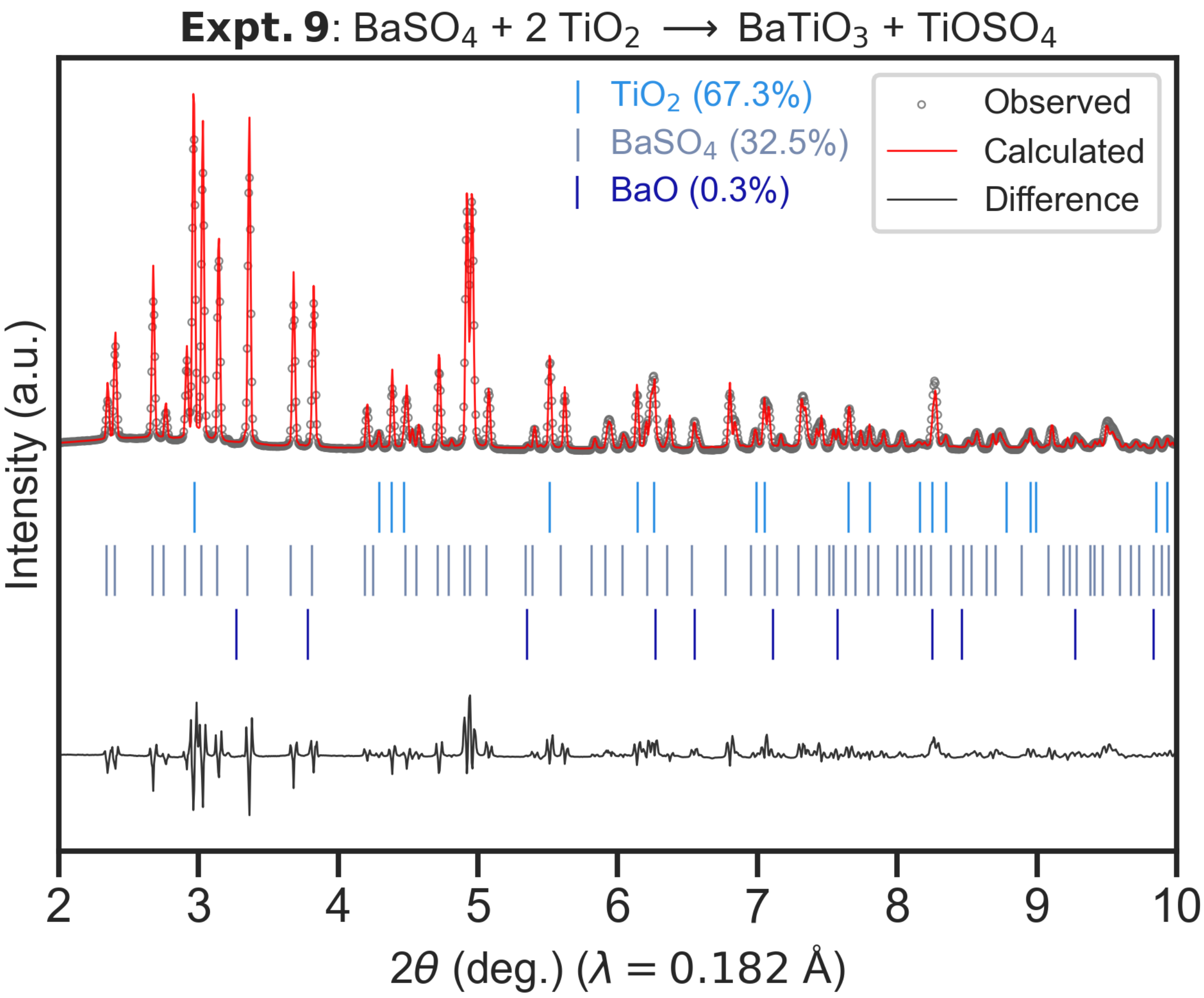

Figure S12: Selected Rietveld refinement for Experiment 9. The observed pattern represents *ex post facto* SPXRD data captured following reaction at T = 594 °C. This value corresponds to the median temperature studied since $BaTiO_3$ was not observed at any temperature. Phase fractions are shown in units of mole percent.

Interface reaction hulls for selected $BaTiO_3$ experiments

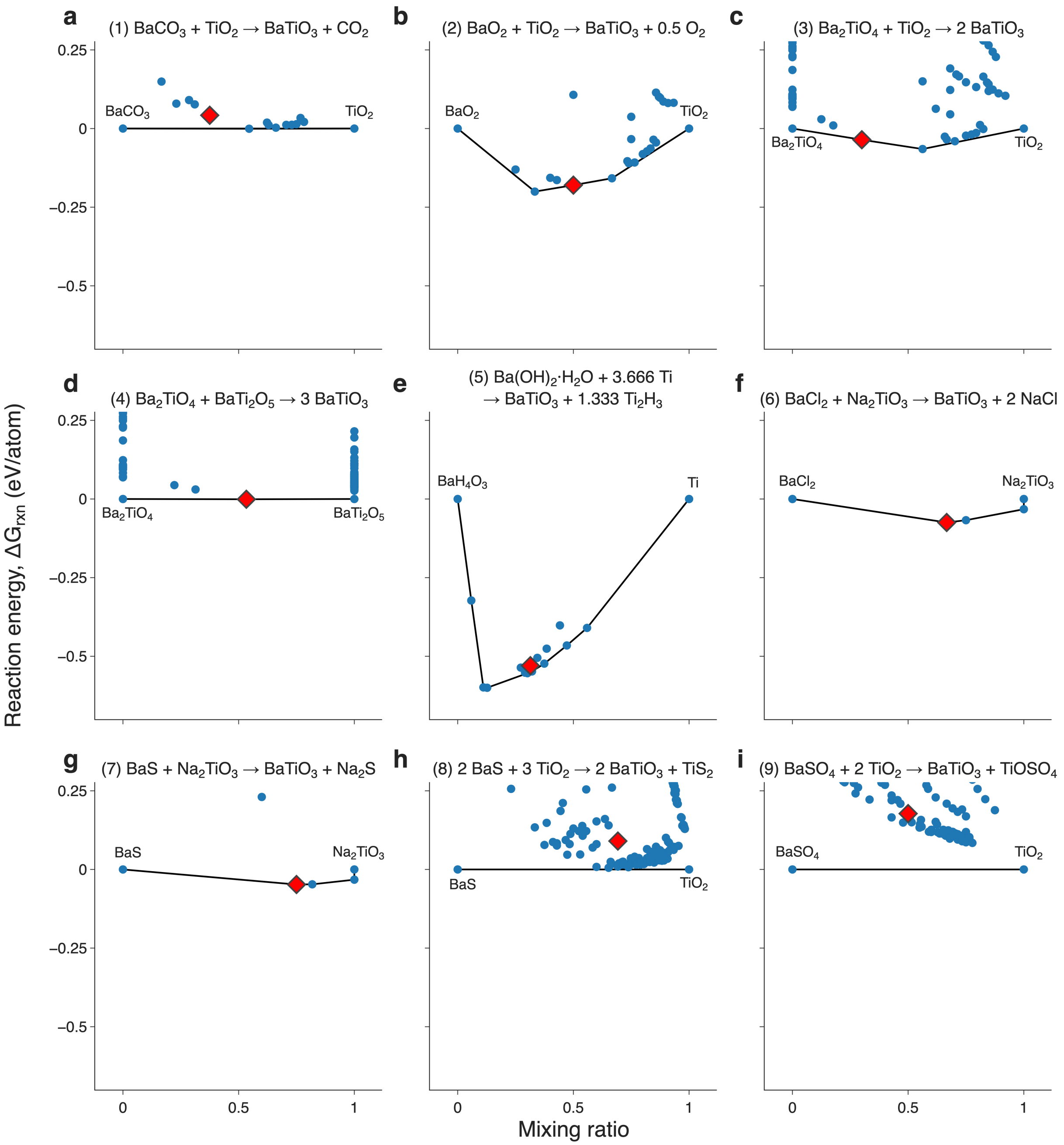

Figure S13: Interface reaction hulls for selected $BaTiO_3$ experiments. (a-i) Hulls for Experiments 1-9, as extracted from the full reaction network calculated during the synthesis planning workflow. Red diamonds mark the selected reactions of interest. All hulls are plotted on a uniform energy scale to facilitate comparison.

All pairwise correlation plots between reaction metrics and experimental outcomes

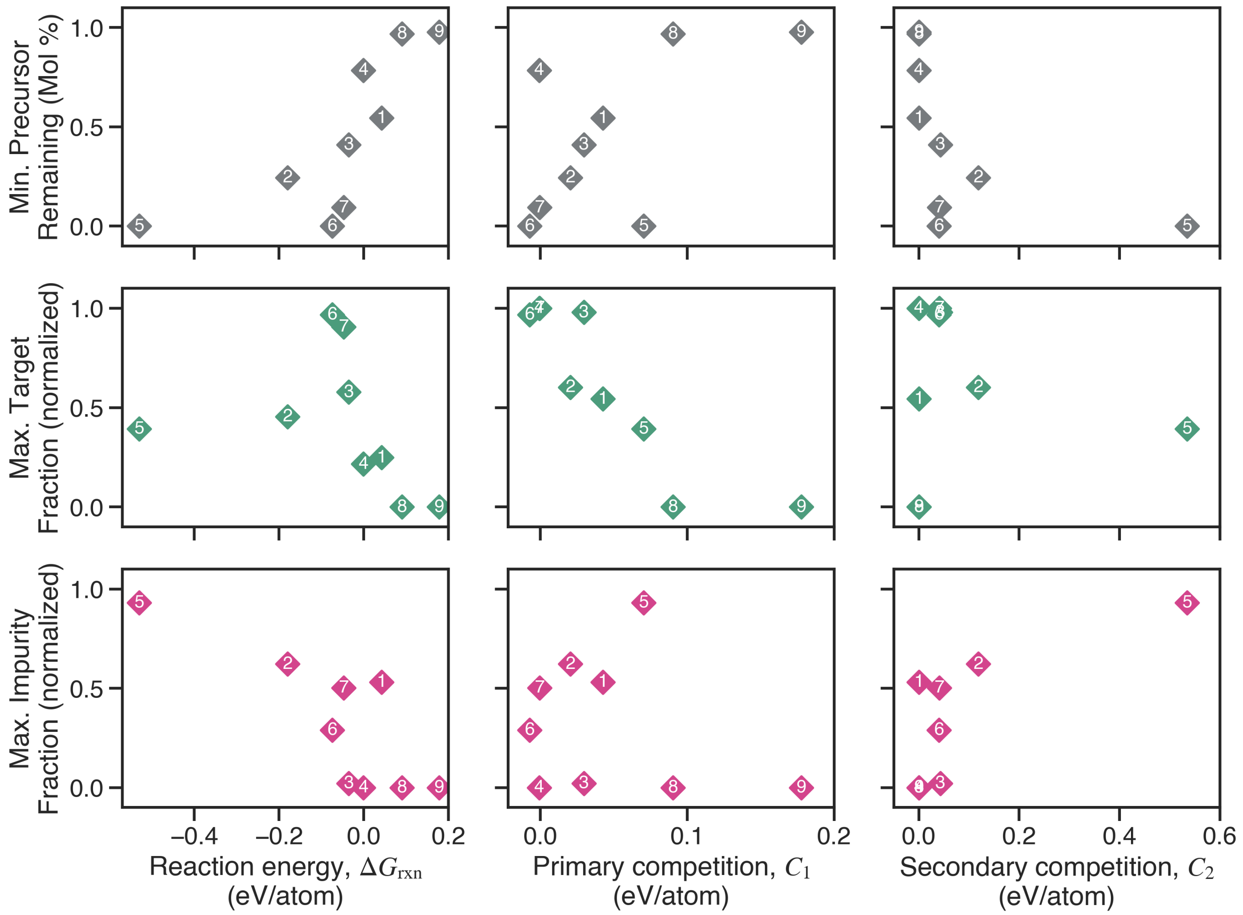

Figure S14: Pairwise correlation plots between reaction metrics and experimental outcomes. Plots of minimum remaining precursor (P), maximum target (T), and maximum impurity (I) formed as a function of reaction energy, primary competition, and secondary competition. The target and impurity plots have been normalized by the amount of precursor consumed ($1 - P$).

Correlations between selectivity metrics

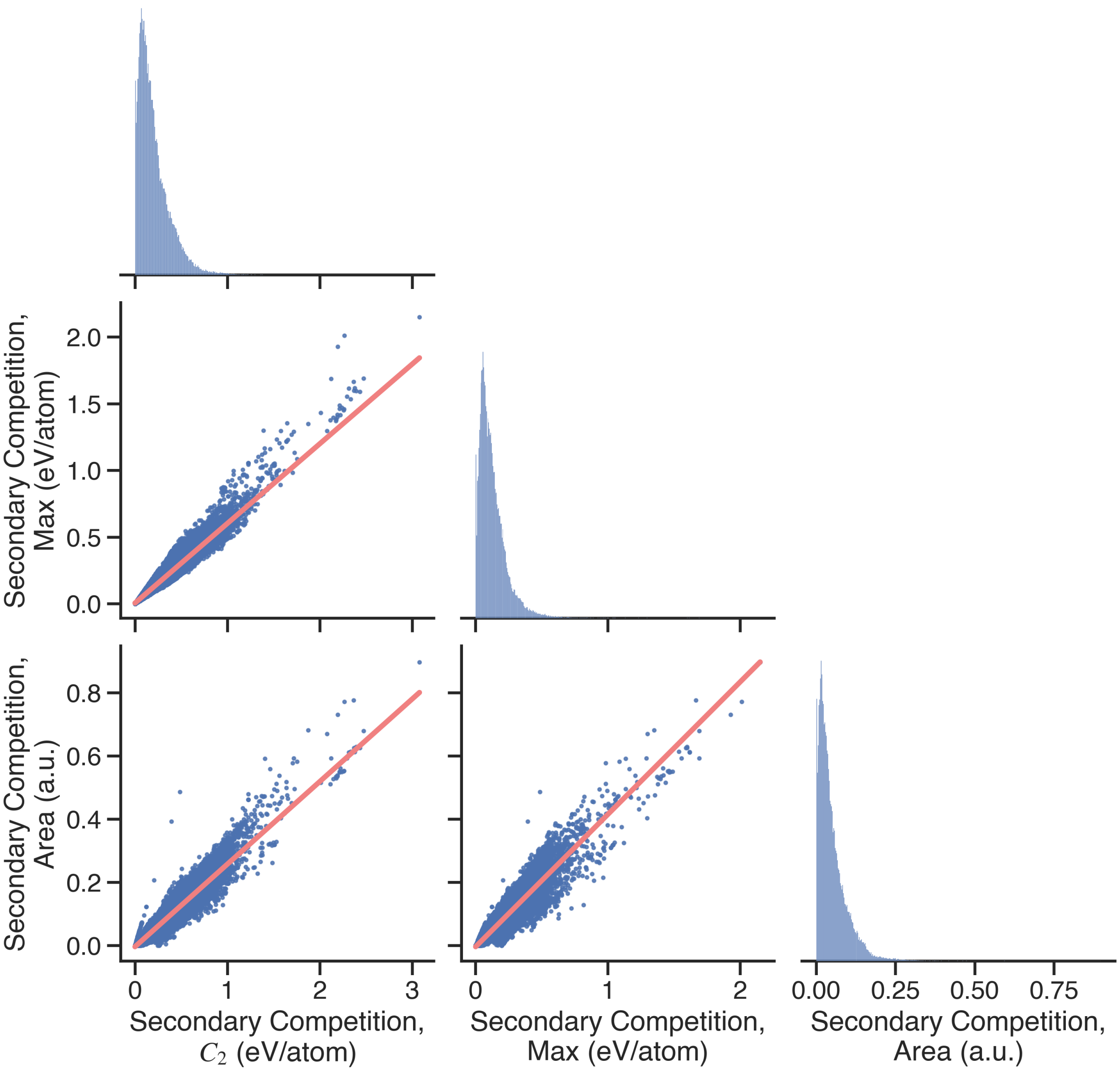

Figure S15: Pairwise correlations of alternative secondary competition metrics. Secondary competition (max) is defined as the sum of the energies of only the secondary reactions with the highest driving forces on either side of the target. Secondary competition (area) is the enclosed area of the interface reaction hull.

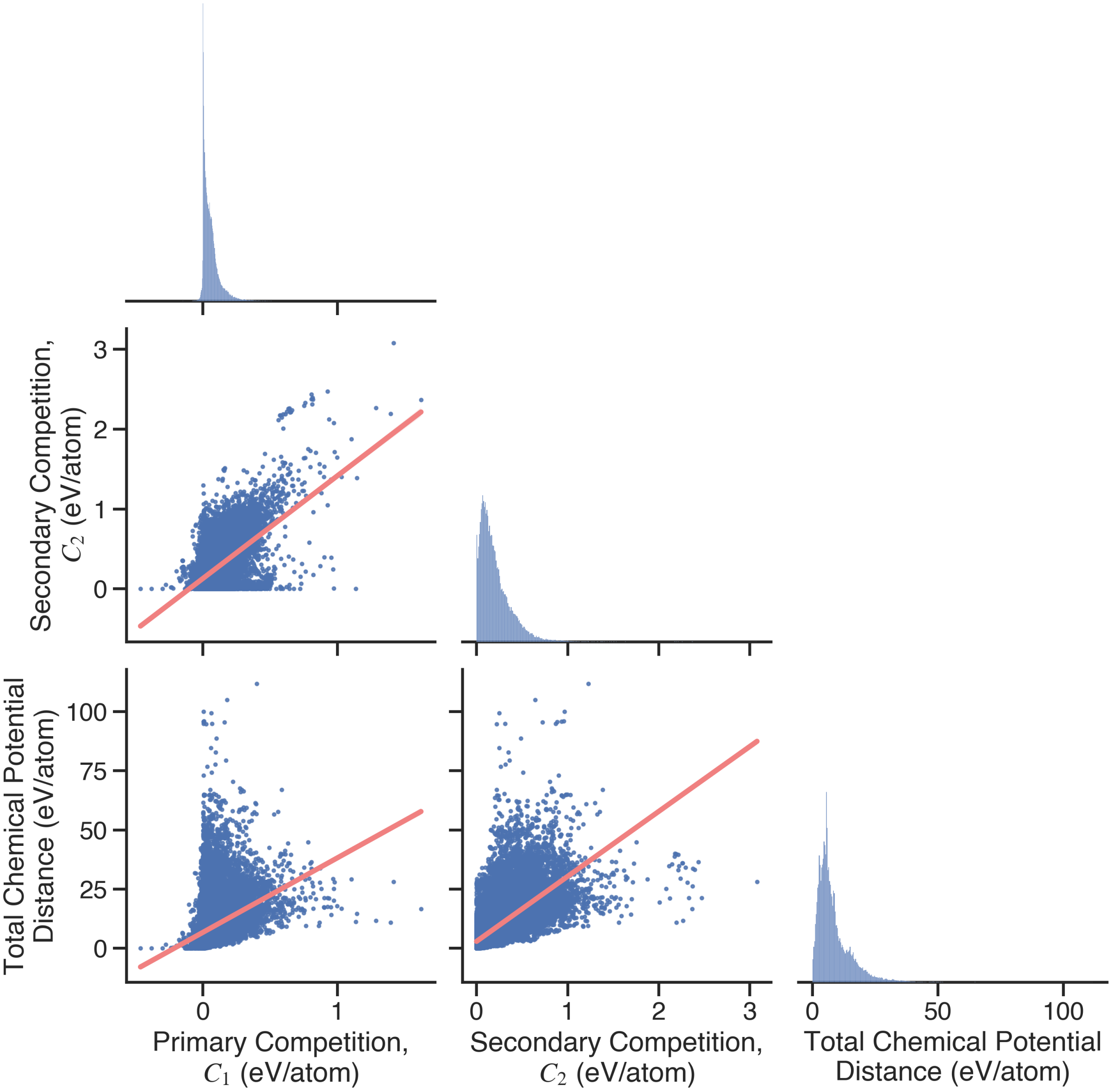

Figure S16: Pairwise correlations of selectivity metrics. The total chemical potential distance is calculated using the methodology outlined in Ref. S1.

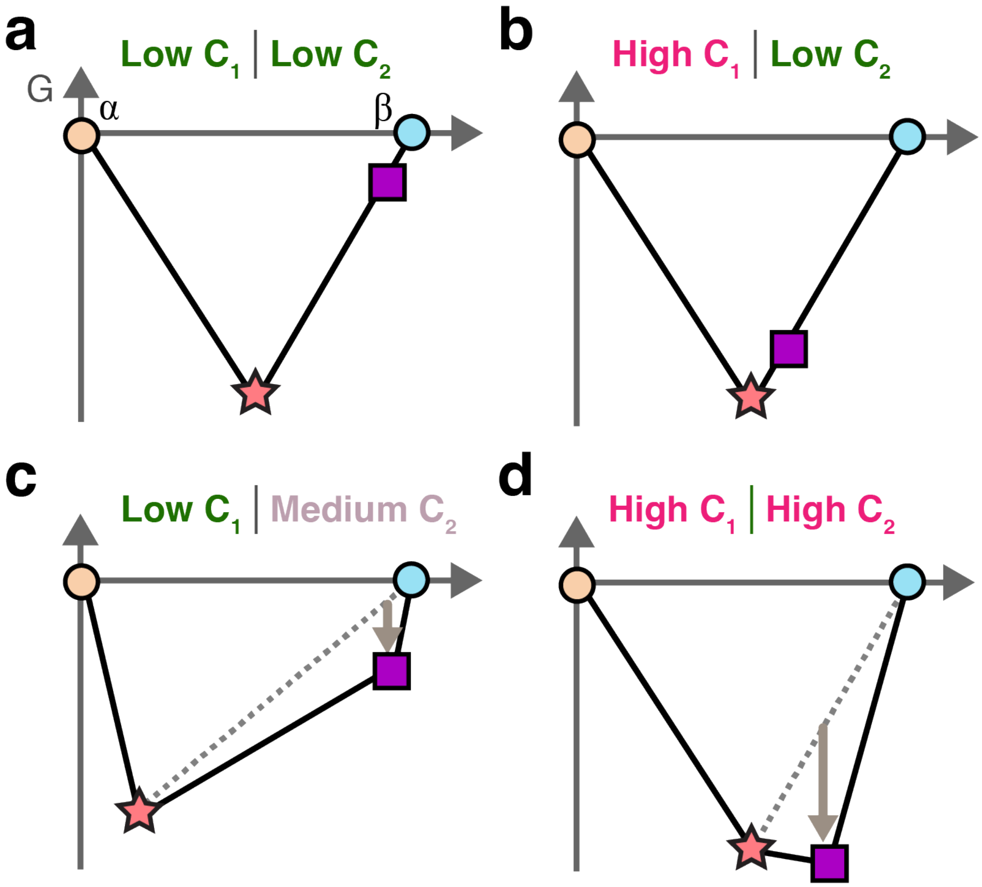

Figure S17: Examples of varying C_1 and C_2 indicating their partial correlation. (a, b) When secondary competition (C_2) is low, primary competition (C_1) can either be low or high. c) However, when C_1 is low, C_2 can not be high due to the geometric constraints of the hull. d) There are no restrictions for both C_1 and C_2 to be high.

Formation energy correction for carbonates

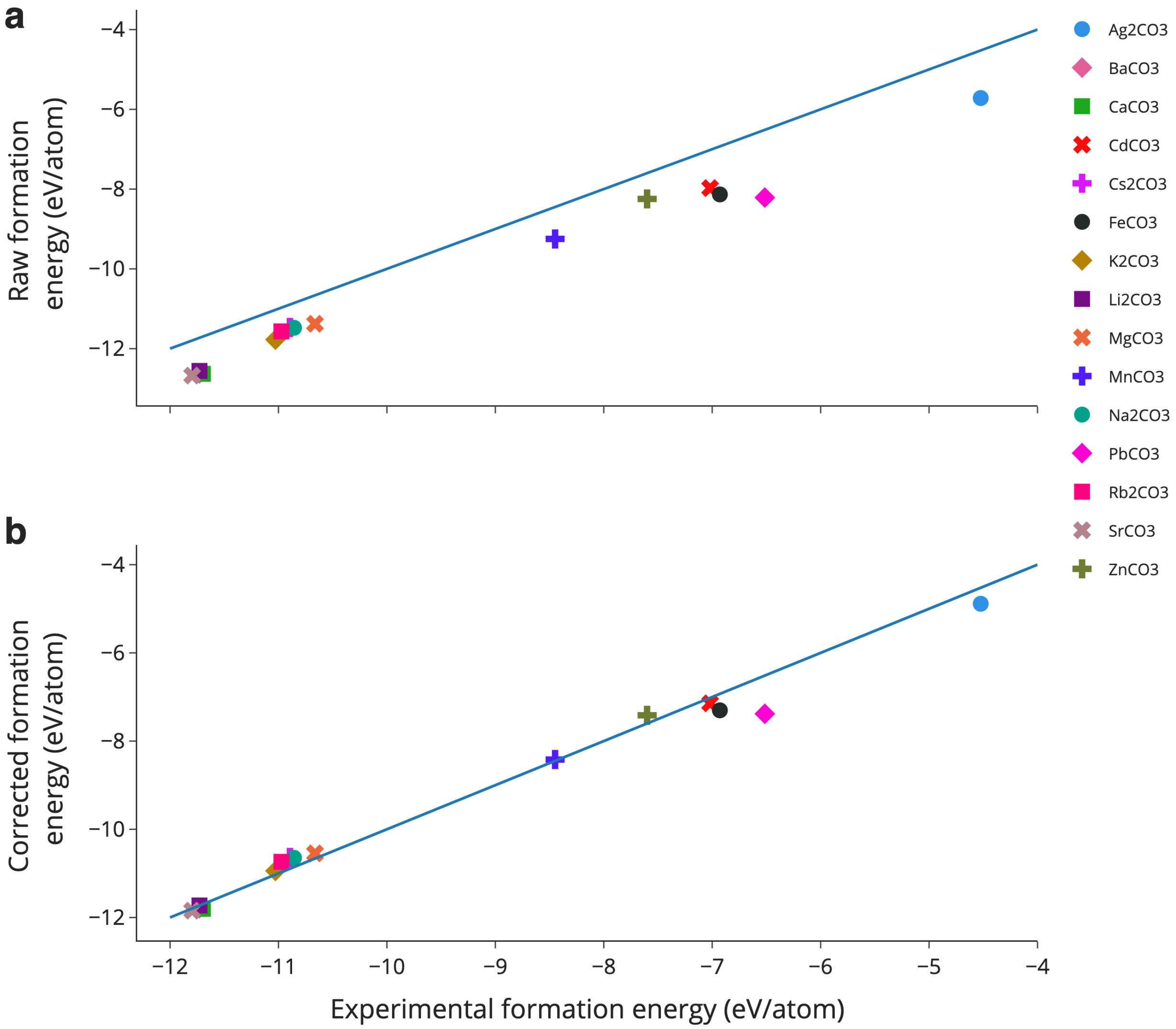

Figure S18: Fitting procedure for formation energy correction of carbonate compounds. (a) Carbonates exhibit a systematic negative shift in predicted Gibbs free energy of formation, ΔG_f (T =300 K) compared to the experimental values. (b) Energies of the same carbonate compounds after applying a fit energy correction of 0.830 eV/CO_3^{2-}.

Gradient furnace setup, heating, and calibration

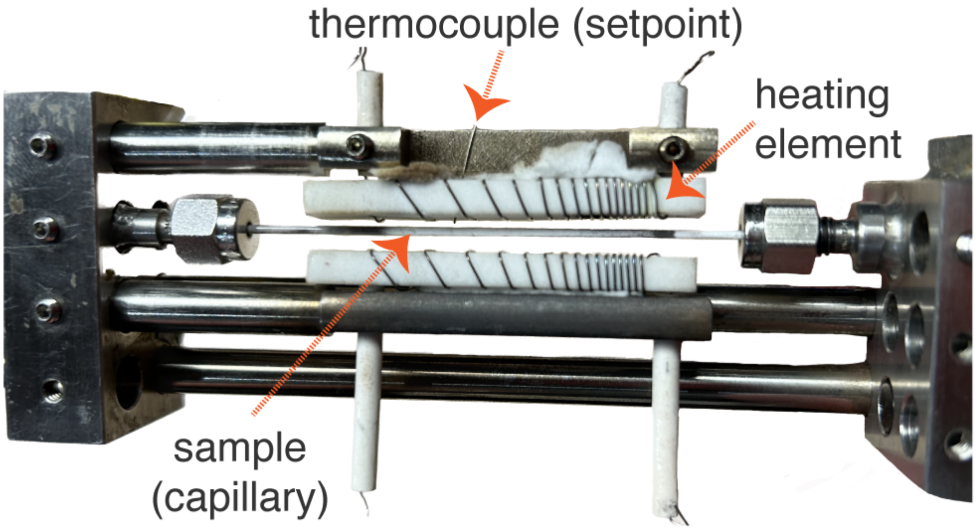

Figure S19: The gradient furnace used in all *ex post facto* SPXRD experiments. The pictured device has the same specifications as described in Ref. S2. The heating wires are wound with variable pitch, resulting in a wide temperature profile over the powder sample (capillary tube). A thermocouple is used as a setpoint for determining the power supplied to the heating elements.

Table S2: Reaction times for selected $BaTiO_3$ experiments. All times are shown in MM:SS format (minutes/seconds). Temperatures correspond to gradient furnace setpoints of T_H = 550 ℃, T_{L1} = 450 ℃, and T_{L2} = 400 ℃.

Expt.	Precursors	Heat	Hold	Cool	Total	Temp.
**1**	$BaCO_3$-TiO_2	09:08	43:28	14:05	66:41	T_H
**2**	BaO_2-TiO_2	01:47	64:38	13:00	79:25	T_{L1}
**3**	Ba_2TiO_4-TiO_2	06:44	18:22	11:48	36:54	T_H
**4**	Ba_2TiO_4-$BaTi_2O_5$	12:07	14:30	12:35	39:12	T_H
**5**	$Ba(OH)_2{\cdot}H_2O$-Ti	07:15	58:30	14:33	80:18	T_{L1}
**6**	$BaCl_2$-Na_2TiO_3	01:36	65:04	22:43	89:23	T_{L2}
**7**	BaS-Na_2TiO_3	02:14	61:26	28:16	91:56	T_{L1}
**8**	BaS-TiO_2	09:03	14:24	11:46	35:13	T_H
**9**	$BaSO_4$-TiO_2	07:15	25:54	13:46	46:55	T_{L1}

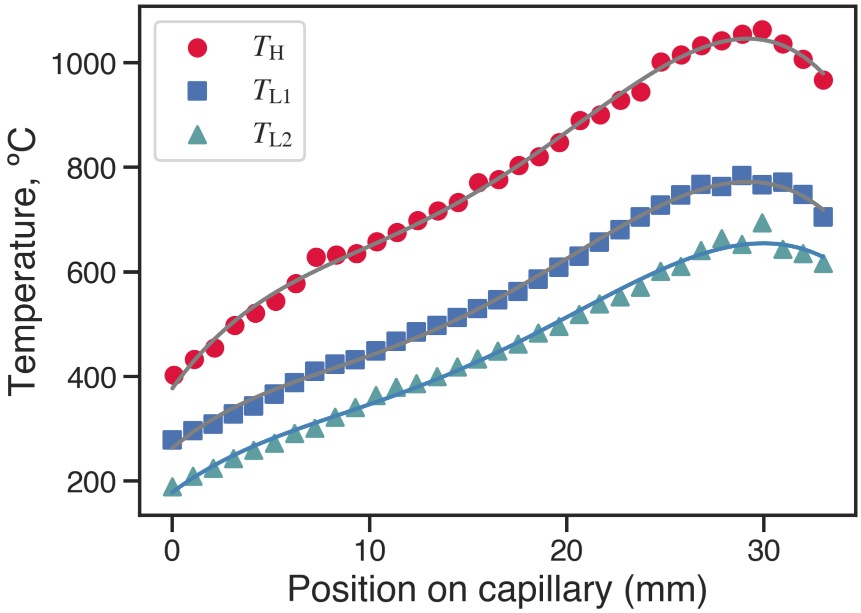

Figure S20: Measured temperature distributions along the length of sample capillary. Temperatures were determined by refinement of lattice parameters from NaCl/Si (T_{L1}, T_{L2}) or Al_2O_3/MgO standards (T_{H}). The calibration curves correspond to gradient furnace setpoints of $T_H = 550$ °C, $T_{L1} = 450$ °C, and $T_{L2} = 400$ °C.

References

(S1) Todd, P. K.; McDermott, M. J.; Rom, C. L.; Corrao, A. A.; Denney, J. J.; Dwaraknath, S. S.; Khalifah, P. G.; Persson, K. A.; Neilson, J. R. Selectivity in Yttrium Manganese Oxide Synthesis via Local Chemical Potentials in Hyperdimensional Phase Space. *Journal of the American Chemical Society* **2021**, *143*, 15185–15194.

(S2) O'Nolan, D.; Huang, G.; Kamm, G. E.; Grenier, A.; Liu, C.-H.; Todd, P. K.; Wustrow, A.; Tran, G. T.; Montiel, D.; Neilson, J. R.; Billinge, S. J. L.; Chupas, P. J.; Thornton, K. S.; Chapman, K. W. A Thermal-Gradient Approach to Variable-Temperature Measurements Resolved in Space. *Journal of Applied Crystallography* **2020**, *53*, 662–670.
