# Supplementary material for: Assessing Thermodynamic Selectivity of Solid-State Reactions for the Predictive Synthesis of Inorganic Materials
Source: ACS Cent Sci. 2023 Oct 16;9(10):1957–75. doi: 10.1021/acscentsci.3c01051 (PMC10604012; doi:10.1021/acscentsci.3c01051)
Supplement: Supplementary file 8 — oc3c01051_si_008.pdf [file oc3c01051_si_008.pdf]

oc-2023-01051z.R1

Name: Peer Review Information for "Assessing Thermodynamic Selectivity of Solid-State Reactions for the Predictive Synthesis of Inorganic Materials"

First Round of Reviewer Comments

Reviewer: 1

Comments to the Author

This article presents a comprehensive method for designing and predicting synthesis routes for inorganic chemistry, with a particular focus on utilizing Solid-State Reactions. The paper is well-written and exhibits a logical flow of information. The main contribution of this work lies in its ability to pave the way for the "retrosynthesis" moment in the synthesis of inorganic compounds, making it a valuable addition to the field.

The authors begin by conducting a thorough data and thermodynamic analysis of the phase competition, providing a solid foundation for their proposed method. Additionally, they make effective use of existing experimental literature, further strengthening their approach. The method is then validated through actual experiments, demonstrating its maturity and applicability.

Reading this paper has been a joy, as it provides valuable insights into the design and prediction of synthesis routes in inorganic chemistry. Based on the strength of its contribution and the quality of its execution, I highly recommend accepting this paper as it is.

Reviewer: 2

Comments to the Author

The manuscript by M. J. McDermott and co-workers provides a new theoretical framework aimed at predicting the best reaction pathways toward a desired oxide solid phase. The suggested framework uses reaction interface metrics that rank various reaction pathways based on their thermodynamic feasibility and selectivity with respect to the formation of target and competing impurity phases. In addition, the manuscript includes a detailed experimental study of the BaTiO3 synthesis, as a case study, using

synchrotron powder diffraction to characterize a pathway for the nine selected synthesis reactions. This manuscript addresses some of the key challenges of predictive synthesis of inorganic materials and would be a valuable resource for solid-state chemists, especially the ones interested in the synthesis of ternary oxides. Therefore, I can recommend the publication of this manuscript after minor revision to address the questions listed below:

- Consider including a comment on the applicability of the suggested computational framework to other, non-oxide chemistries as well as other synthesis routes, particularly those that include liquid phase (flux growth) and thus are likely nucleation-limited.

-The evaluation of a hundred reactions to find a synthesis “recipe” for one compound as a way to test the predictive power of the suggested computational framework as well as to show its limitations is certainly justified for this study. However, if the intention is to aid experimentalists in their pursuit of optimized synthesis conditions, I would encourage authors to consider creating a user-friendly database/portal that includes reaction networks to produce selected ternary oxides, where sensible constraints are implemented for the selection of precursor phases. In addition to limiting the choice of precursor elements (15+3 in the case of BaTiO3 example), the complex precursors have to be eliminated, e.g. those that are not available commercially, or only theoretically predicted, or not safe to handle, or whose synthesis involves multiple steps, etc. Using BaTiO3 synthesis as an example, it is unlikely that explosive and volatile Ti(ClO4)4 and highly reactive Ba6Mg23 are sensible choices for the synthesis of BaTiO3. There are more examples of “extremely unpractical” precursors suggested for BaTiO3 synthesis in Table 3. It is valuable to consider this and similar reactions when testing the proposed computational framework, however with regard to the experimental realization, such reactions have very little value. By putting certain constraints on the selection of precursors, the manageable size of the reaction networks can be (potentially) evaluated for a wider selection of ternary oxides.

-- In addition to SI file, additional files were provided, e.g. tables with several thousand of predicted reactions or reactions extracted from the literature. The files are in pdf format and are difficult to interpret because the tables appear to split during conversion from XLSL files. Please ensure that the supplied files are XLSL format.

- In the Precursor Materials section, consider including a comment regarding how pure the precursors were from PXRD. This is especially important for the precursors that are synthesized in this work using previously published methods, e.g. BaS, BaSO4, BaTi2O4, BaTi2O5 and Na2TiO3.

Author's Response to Peer Review Comments:

Authors’ response to reviewer comments

We thank the reviewers for their very kind and constructive comments. With the following revisions, we hope that our manuscript will be acceptable for publication. Please find our point-by-point response to the reviewers’ comments below. The text has been marked following these guidelines:

Original reviewers' comments: *italicized, black*
Authors' responses: blue
Quoted changes to the manuscript: highlighted yellow

Reviewer(s)' Comments to Author:

Reviewer: 1

Recommendation: Publish in ACS Central Science without change.

Comments:
This article presents a comprehensive method for designing and predicting synthesis routes for inorganic chemistry, with a particular focus on utilizing Solid-State Reactions. The paper is well-written and exhibits a logical flow of information. The main contribution of this work lies in its ability to pave the way for the "retrosynthesis" moment in the synthesis of inorganic compounds, making it a valuable addition to the field.

The authors begin by conducting a thorough data and thermodynamic analysis of the phase competition, providing a solid foundation for their proposed method. Additionally, they make effective use of existing experimental literature, further strengthening their approach. The method is then validated through actual experiments, demonstrating its maturity and applicability.

Reading this paper has been a joy, as it provides valuable insights into the design and prediction of synthesis routes in inorganic chemistry. Based on the strength of its contribution and the quality of its execution, I highly recommend accepting this paper as it is.

We thank the reviewer for their positive feedback and very kind words. We are delighted to be able to share this work with the scientific community and are excited for future developments toward developing and achieving retrosynthesis of inorganic materials!

Reviewer: 2

Recommendation: Publish in ACS Central Science after minor revisions noted.

Comments:

The manuscript by M. J. McDermott and co-workers provides a new theoretical framework aimed at predicting the best reaction pathways toward a desired oxide solid phase. The suggested framework uses reaction interface metrics that rank various reaction pathways based on their thermodynamic feasibility and selectivity with respect to the formation of target and competing impurity phases. In addition, the manuscript includes a detailed experimental study of the BaTiO3 synthesis, as a case study, using synchrotron powder diffraction to characterize a pathway for the nine selected synthesis reactions. This manuscript addresses some of the key challenges of predictive synthesis of inorganic materials and would be a valuable resource for solid-state chemists, especially the ones interested in the synthesis of ternary oxides. Therefore, I can recommend the publication of this manuscript after minor revision to address the questions listed below:

We thank the reviewer for their positive recommendation and are happy to address their questions below.

- Consider including a comment on the applicability of the suggested computational framework to other, non-oxide chemistries as well as other synthesis routes, particularly those that include liquid phase (flux growth) and thus are likely nucleation-limited.

This is a helpful suggestion to improve the manuscript. In this work, we primarily study oxide chemistries due in part to their prevalence and technological importance in the scientific literature. However, we have no reason to suspect that our reaction selectivity framework would be unsuitable for other chemistries where solid-state synthesis is currently used, as the fundamental assumptions of our approach still hold (i.e., most reactions are between crystalline solid-solid interfaces being heated). These additional chemistries include those of most ionic compounds: halides, other chalcogenides, pnictides, some silicides/carbides/hydrides, as well as combinations of these (e.g., polyatomic ions), and complex compounds (3+ elements). There are some additional considerations, however, that should be accounted for when working with other chemistries. For our framework, we suspect the most impactful of these is the **availability of thermodynamic data** due to bias towards the inclusion of oxides. For example, in the Materials Project database, 81,877 out of 154,718 compounds (53%) contain oxygen. While our computed databases continue to be improved and diversified with enumeration of additional structures, the prediction of phase competition in *oxide* systems will likely remain more accurate over other chemistries, simply because we have a more thorough description of the phase space.

Regarding the applicability of our approach to other synthesis methods (e.g., flux synthesis), it is likely that our framework will be less applicable, but still informative/useful. In particular, the use

of reaction free energy (ΔG_{rxn}) and primary competition (C_1) relies on very few assumptions about the nature of the precursors, as both assess the advantage of the target reaction using only its total driving force; in general, minimizing both of these metrics would be favorable for any synthesis approach. The secondary competition (C_2), on the other hand, assumes the prolonged existence of precursor-impurity interfaces, which seem to be more unique to solid-state powder synthesis. The applicability of these metrics to other synthesis methods would be a good topic for future studies.

We added a comment to the manuscript reflecting some important points of the above discussion, and thank the reviewer for their suggestion.

Page 34-35: Although our current study focuses on the synthesis of oxides, we expect our synthesis planning approach to be suitable to other chemistries where solid-state synthesis can be employed. This includes the chemistries of most ionic compounds: halides, chalcogenides, pnictides, some silicides/carbides, etc. Still, one must ensure that there is enough thermodynamic data available to accurately model phase competition in the chemical system of interest. This is generally true for oxide compounds due to their high prevalence in literature and thermodynamic data; as one example, at present, ~53% of the nearly one-hundred and fifty-thousand compounds in the Materials Project contain oxygen. While the predictive accuracy is currently greatest for oxides, we expect our approach to grow in accuracy and general applicability as computed materials databases grow in size and chemical complexity.

- The evaluation of a hundred reactions to find a synthesis "recipe" for one compound as a way to test the predictive power of the suggested computational framework as well as to show its limitations is certainly justified for this study. However, if the intention is to aid experimentalists in their pursuit of optimized synthesis conditions, I would encourage authors to consider creating a user-friendly database/portal that includes reaction networks to produce selected ternary oxides, where sensible constraints are implemented for the selection of precursor phases. In addition to limiting the choice of precursor elements (15+3 in the case of BaTiO3 example), the complex precursors have to be eliminated, e.g. those that are not available commercially, or only theoretically predicted, or not safe to handle, or whose synthesis involves multiple steps, etc. Using BaTiO3 synthesis as an example, it is unlikely that explosive and volatile Ti(ClO4)4 and highly reactive Ba6Mg23 are sensible choices for the synthesis of BaTiO3. There are more examples of "extremely unpractical" precursors suggested for BaTiO3 synthesis in Table 3. It is valuable to consider this and similar reactions when testing the proposed computational framework, however with regard to the experimental realization, such reactions have very little value. By putting certain constraints on the selection of precursors, the manageable size of the reaction networks can be (potentially) evaluated for a wider selection of ternary oxides.

This is a great point, and we understand that the typical unfiltered workflow outputs (e.g., ~83K reactions for $BaTiO_3$) may be manually unparseable for the average user. The reviewer's suggestion is addressed within our provided Python code (*reaction-network* package at https://github.com/materialsproject/reaction-network), which includes functionality for filtering a set of generated synthesis recipes by a user's available precursors. To make this easier, we also include a list of 586 commonly available precursor compositions compiled from chemical suppliers (e.g., Sigma-Aldrich), available within the package at the file path: *src/rxn_network/data/available_precursors.json.* This serves as a convenient starting point for filtering and downselecting possible recipes to try in the lab, and the precursor list can be adjusted according to what precursors are accessible. With the smaller list of filtered recipes, it is much easier to manually select a synthesis recipe that is appropriate for one's experimental facilities.

To demonstrate this, we used the aforementioned list of available precursors to filter the $BaTiO_3$ synthesis recipes generated in this work. This reduces the number of "closed" (non-open) BaTiO3 reactions from 82,985 to a more manageable 478 recipes. The resulting filtered tables for both closed and open reactions have been provided as additional XLSX in the Supporting Information. Additionally, the manuscript text has been updated accordingly to reflect these capabilities and the new SI files:

Page 26: In our provided code (see Methods), we support functionality for the former by including a list of hundreds of common precursors compiled from the catalogs of chemical suppliers. Filtering by these commonly available precursors (e.g., BaCO3, Ba3(PO4)2, TiO2) reduces the full set of 82,985 BaTiO3 synthesis reactions to 478, making the generated recipes more easily parseable and readily testable. The filtered BaTiO3 reactions, including the corresponding open-O2 reactions, are provided in the Supporting Information. While filtering by conventional precursors is practically convenient, we consider the unorthodox nature of the unfiltered reactions an advantage of our approach, as this permits synthesis recommendations that expand beyond traditional chemical intuition. Still, synthesis recipes must be screened for reactivity, volatility, safety, and material costs. These challenges can be mitigated through the use of additional data or models; for example, reactivity can be approximated through surrogate data, such as defect formation energies or physical properties (melting points, hardness, etc.).

Page 44:

- Table of 478 predicted/ranked closed BaTiO3 synthesis reactions filtered by commonly available (i.e., purchasable) precursors (XLSX)
- Table of 622 predicted/ranked open-O2 BaTiO3 synthesis reactions filtered by commonly available (i.e., purchasable) precursors (XLSX)

In the added text, we note that while filtering by conventional precursors offers a more convenient recipe selection to choose from, it is counter to one of the main takeaways of this work, which is that *unconventional* precursors (particularly those of different chemistries) are often capable of offering a greater thermodynamic advantage than off-the-shelf precursors. Furthermore, what is considered sensible in today's understanding of synthesis does not necessarily mean *optimal*; for example, we showed that the reaction of BaS+Na_2TiO_3 outperforms the standard $BaCO_3$+TiO_2 approach despite being unsensible at first glance. However, we understand that there is a careful balance here that depends on the particular user, and filtering by available precursors is also a convenient approach because it removes many theoretical and/or dangerous chemicals. In the future, we are interested in developing a ranking approach for the predicted safety of a synthesis recipe; however, this requires a significant amount of additional data.

We agree with the reviewer that the creation of a database/portal for synthesis recipes is ideal for user experience. Because doing so requires a large number of calculations, careful data curation, and possibly front-end web development, we are not able to offer this at the moment; however, this is a goal for the Materials Project website in the upcoming year. In the meantime, the *reaction-network* package is well-documented, supported, and tested, which should make our framework more broadly accessible to the community.

-- In addition to SI file, additional files were provided, e.g. tables with several thousand of predicted reactions or reactions extracted from the literature. The files are in pdf format and are difficult to interpret because the tables appear to split during conversion from XLSL files. Please ensure that the supplied files are XLSL format.

We are sorry to hear that the supporting data tables were provided in a PDF format. We can confirm that these files were uploaded in the XLSX format during submission. According to our submission portal, the original XLSX files should be available to download by clicking the name of the file. Regardless of how the issue arose, we will work with the editorial office to ensure that the uploaded files are provided in XLSX format with publication.

- In the Precursor Materials section, consider including a comment regarding how pure the precursors were from PXRD. This is especially important for the precursors that are synthesized in this work using previously published methods, e.g. BaS, BaSO4, BaTi2O4, BaTi2O5 and Na2TiO3.

We have updated the *Precursor Materials* section in the Methods to include a discussion of the purities of the four synthesized precursors (Ba_2TiO_4, $BaTi_2O_5$, BaS, and Na_2TiO_3) according to our

laboratory PXRD data. Please see the updated manuscript text reproduced below. For better context, we have included the entire paragraphs (i.e., with the existing accompanying text).

Pages 41-42: Precursors prepared via solid-state synthesis include barium orthotitante (Ba_2TiO_4), $BaTi_2O_5$, barium sulfide (BaS), and sodium metatitanate (Na_2TiO_3). Phase purities were assessed via laboratory powder x-ray diffraction (PXRD) analysis performed with a Bruker D8 Discover diffractometer using Cu Kα radiation.

Ba_2TiO_4 was prepared using stoichiometric amounts of $BaCO_3$ and anatase TiO_2. The chemicals were mixed, ground using a mortar and pestle, placed in an alumina boat inside of a mullite process tube with self-sealing endcaps, and then heated at 950 ºC for 16 hrs under Ar flow with a heating rate of 10 ºC/min. The powder was then reground and reheated at 1100 ºC for another 16 hrs at a heating rate of 10 ºC/min. Handling operations were completed in an Ar glovebox due to the hygroscopic nature of Ba_2TiO_4. The product was phase pure β-Ba_2TiO_4 with no observed impurities.

$BaTi_2O_5$ was prepared using stoichiometric amounts of $BaCO_3$ and anatase TiO_2. The chemicals were mixed, ground using a mortar and pestle, and heated in an alumina boat at 900 ºC for 5 hrs as a pre-treatment step. The powder was then reground and reheated at 1220-1225 ºC for 24 hrs with heating and cooling steps of 3 hrs. The product was mostly phase pure with minor impurities, including a small amount of unreacted $BaCO_3$ precursor (<3 mol %) and $Ba_6Ti_{17}O_{40}$ (~3 mol %). The latter phase was similarly observed in Ref. 49, where its formation was attributed to the thermodynamic instability of $BaTi_2O_5$ at temperatures outside a very narrow range (1220-1230 ºC).

BaS was prepared using $BaSO_4$ and activated carbon (C, J.T.Baker 99.9%). The chemicals were mixed, ground using a mortar and pestle, pressed into a 0.5” diameter pellet with two tons of force, and heated in an alumina boat at 1100 ºC for 7-10 min in air, with a heating rate of 10 ºC/min and natural cooling in the furnace. The product was phase pure with no detectable impurities.

Na_2TiO_3 was prepared using stoichiometric amounts of sodium hydroxide (NaOH, Fisher Scientific 99.9%) and anatase TiO_2, with a slight excess of NaOH. The chemicals were mixed, ground using a mortar and pestle, and heated in an alumina boat at 500 ºC for 2 hrs with a heating rate of 10 ºC/min. The product was mostly phase pure with minor impurities. The sodium titanate peaks are best fit by a cubic α-Na_2TiO_3 structure with a small crystallite size. A minor amount of unreacted anatase TiO_2 was present in the product (~1 mol %). Na_2CO_3 also appears to be present

as an impurity (~11 mol %); we suspect this is due to contamination of the NaOH precursor via reaction with CO2 in the air.

Note that the purities of all purchased precursors (including the $BaSO_4$ mentioned by the reviewer) are currently provided in the text.

In general, we found that our synthesized precursors were either phase pure or containing only minor impurities. Specifically, most impurities were unreacted precursor(s) at small concentrations (1-3 mol%), which should not significantly affect our experimental analysis. For the Na2TiO3 precursor, however, our follow-up PXRD analysis showed that Na2CO3 was present. We found this to be a helpful observation, as it provides further context and rationale for our existing discussion on the presence of carbonate impurities, Ba3(CO3)Cl4 and BaCO3, seen in Experiment 6. We updated the manuscript with some additional text describing how this impurity may have played a role in the reaction pathway:

Page 30: Unexpectedly, the dominant impurities in Expt. 6 are carbonate compounds: Ba3(CO3)Cl4, and BaCO3. We presume this results from minor contamination of the precursors via reaction with CO2 in the air**;** some Na2CO3 observed in the precursor (see Methods) may have also contributed to the formation of the barium carbonate impurities via energetically favorable Ba/Na ion exchange reactions.

Overall, we believe that the additional precursor purity analysis has strengthened the quality of our discussion, and thank the reviewer for their suggestion.
